# The Enzyme Portal: a case study in applying user-centred design methods in bioinformatics

**DOI:** 10.1186/1471-2105-14-103

**Published:** 2013-03-20

**Authors:** Paula de Matos, Jennifer A Cham, Hong Cao, Rafael Alcántara, Francis Rowland, Rodrigo Lopez, Christoph Steinbeck

**Affiliations:** 1 EMBL-EBI, Wellcome Trust Genome Campus, Hinxton, Cambridge, CB10 1SD, UK; 2 European Commission, European Research Council Executive Agency, (Formally of EMBL-EBI), Brussels, Belgium

**Keywords:** 3D protein structure, Biological pathways, Card sorting, Design, Enzyme, Enzyme portal, Implementation, Personae, Prototyping, User-centered design (USA spelling), User-centred design, User experience, User profiles, User requirements, Usability testing

## Abstract

User-centred design (UCD) is a type of user interface design in which the needs and desires of users are taken into account at each stage of the design process for a service or product; often for software applications and websites. Its goal is to facilitate the design of software that is both useful and easy to use. To achieve this, you must characterise users’ requirements, design suitable interactions to meet their needs, and test your designs using prototypes and real life scenarios.

For bioinformatics, there is little practical information available regarding how to carry out UCD in practice. To address this we describe a complete, multi-stage UCD process used for creating a new bioinformatics resource for integrating enzyme information, called the Enzyme Portal (http://www.ebi.ac.uk/enzymeportal). This freely-available service mines and displays data about proteins with enzymatic activity from public repositories via a single search, and includes biochemical reactions, biological pathways, small molecule chemistry, disease information, 3D protein structures and relevant scientific literature.

We employed several UCD techniques, including: persona development, interviews, ‘canvas sort’ card sorting, user workflows, usability testing and others. Our hope is that this case study will motivate the reader to apply similar UCD approaches to their own software design for bioinformatics. Indeed, we found the benefits included more effective decision-making for design ideas and technologies; enhanced team-working and communication; cost effectiveness; and ultimately a service that more closely meets the needs of our target audience.

## Background

User-centred design (UCD) is defined as *“an approach to design that grounds the process in information about the people who will use the product. UCD processes focus on users through the planning, design and development of a product”* [Usability Professionals Association http://www.upa.org]. With the aims of UCD in mind, we have designed and built a new digital portal, ‘Enzyme Portal’ to display publicly available enzyme-related information; available at http://www.ebi.ac.uk/enzymeportal. The aim of this freely-available service is to bring together disparate biological and chemical data, so that all the information at a specific point in time about a given enzyme (or other protein with enzymatic activity, such as receptors), can be explored in one place.

In the recent past, integration of bioinformatics data has proven challenging due to the vast amounts available in the public domain, and in some cases due to the lack of agreed data-sharing standards. In spite of these hurdles, much has been published recently on integration software and portals for bioinformatics applications, often aimed at the bench scientist rather than the informatician [1-5] (also see EBI Search, EMBL-EBI’s gene and protein data summary service, example [6], unpublished, which is based on the EB-eye search [7]).

At EMBL-EBI we have recently moved to applying UCD techniques to develop new software services [8] and this article is the first full account of this type of work for bioinformatics. We describe the methodology applied for the design and development of the Enzyme Portal so that others may be inspired to use the same approaches and see the benefits of having a more useful and usable end product.

### UCD for bioinformatics in the literature

It is recognised that bioinformatics resources suffer from significant usability problems. Javahery *et al.*, for example, make the point that bioinformatics interfaces “lack sophistication” compared to those that people come across in their daily lives on other websites and in other software applications [9]. Moreover, Bolchini *et al.*, who performed usability inspections for four major bioinformatics web applications, showed that users were unable to complete their tasks due to usability problems [10]. The result is that user expectations are frequently not met when interfacing with bioinformatics resources, and this influences the rate of adoption and general use of the data and services [9]. This is unfavourable, since the public domain has invested money in creating these resources and data content, so the greatest value possible should be leveraged from them if the funders are to realise a good return on their investment. By applying proven practices in usability engineering we, as a community, have the opportunity to significantly improve the usability, and thus utility, of bioinformatics resources. Indeed, in Rutherford and co-workers’ article, which demonstrated improved usability of one type of gene browser view compared to another, they state: “*it is essential that usability issues are key design criteria for bioinformatics software if it is to be of maximum value to researchers who are increasingly reliant on it*” [11].

In summary, usability is a genuine problem for bioinformatics services. To address it, we believe that users should be taken into account from inception of new bioinformatics resources, rather than at the end when the project is already well under development – with the caveat that heuristic analysis of existing systems may also be helpful, for example, when redesigning a service [12].

### Challenges when applying UCD to bioinformatics

UCD is a general design philosophy, therefore should theoretically be applicable to any domain. For example, it has been successfully applied to the Web for e-commerce [13], mobile development [14] and gaming [15]. However, case studies about how to increase usability of scientific software are in short supply in the literature, and virtually non-existent for bioinformatics, specifically. One example for chemistry, [16], showed how the authors applied UCD to design an e-science laboratory book. They observed chemists in the laboratory and noted how they interacted with their physical laboratory notebook, which then inspired the design of a digital version.

Moreover, the lack of ‘step-by-step’ information for UCD in bioinformatics may be due to the fact that bioinformatics is a complex data domain. Consequently, we suggest that there is a unique *combinatio*n of challenges to be overcome when attempting to apply UCD to bioinformatics; these include:

• ‘Dry’ (computational) and ‘wet’ (lab-based) life science research communities often use the same software resources, so there are challenges to meet their diverse needs, and to account for their different level of computer skills.

• It is difficult to measure the impact of UCD. Desired outcomes of bioinformatics services are improved access and understanding of the data being provided, which in turn, may lead to more fruitful scientific discoveries. ‘Discovery’ is an intangible metric and therefore difficult to demonstrate to stakeholders.

• Historically, this community has not used UCD approaches; it requires a ‘cultural shift’. Bioinformatics applications have often been command-line driven and technical in nature. Moving these types of functionalities to the Web is a new frontier, which sometimes meets resistance when it is perceived that usability practitioners may be ‘over-simplifying’/‘dumbing down’ interfaces.

• Biology is a complex and constantly evolving subject where standard rules often have exceptions, therefore interfaces to the information are difficult to design. Take Enzyme Commission (EC) nomenclature, for example, which is a hierarchical structure of the data to describe enzyme function. This is a set of rules used by some biochemists to categorise enzymes, but it fails to separate all enzymes effectively for other life scientists, who use the same data resources. For instance, COX-1 and COX-2, which have the same EC number (rule), but have very different (scientifically and commercially important) functions in the body. Likewise, criteria to define a ‘small molecule’ or ‘chemical’ fail to account for large biomolecules such as peptides or tRNA.

• Vast amounts of interconnected data are being generated in bioinformatics. It is a huge technical challenge to present the data in meaningful ways to the user. One has to consider the technical constraints and scalability as well as what the user wants and make compromises.

• It is difficult to find individuals with sufficient knowledge of three discrete sciences (namely, human-computer interaction, molecular biology/biochemistry/bioinformatics, and computing) to carry out UCD work effectively in bioinformatics; a problem for many complex domains [17]. This is especially true, since empathy with the end user is key to developing effective strategies for capturing user requirements, designing solutions, and setting plausible scenarios and tasks for usability testing.

To attempt to meet the above challenges, we devised a full lifecycle of UCD techniques to design the Enzyme Portal. For example, we used a bespoke variant of card sorting (‘canvas sort’, described later) to allow prioritisation and categorisation of a large number of data items and functionalities, instead of applying a generic card sorting approach (such as in [18]). Hence, we believe that this case study may be informative for those wishing to design digital services for the life sciences, where complex interconnected data is involved.

## Methods

**Figure 1:**
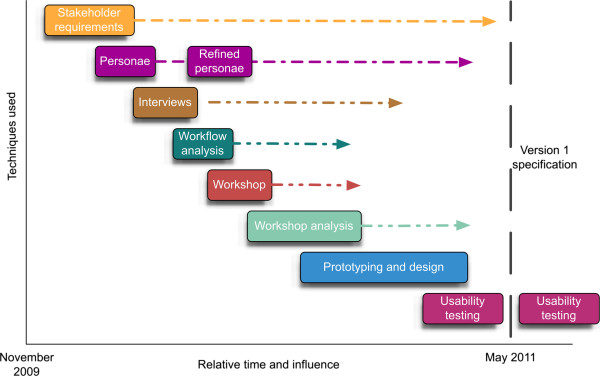
**Outline of the user-centred design process used during the project.** The boxes indicate the approximate time taken to complete each step and the dotted lines indicate the scope of influence they had on the project. Usability testing has continued right through to the interactive prototype. Software implementation started once the first version of the specification was available. Note: ‘personae’ are user profiles; see details in the main article.

A summary of our UCD approach is outlined in Figure 1. Chilana *et al.*[17] have shown that effective results in a UCD process can be achieved when user experience analysts either work closely with domain experts, or are experts in the domain themselves and have had training in usability evaluation. For creating the Enzyme Portal, we used the former approach, where domain experts, such as scientists working in the field of enzymology or pharmaceutical research were consulted at several points in the development process to inform the most appropriate design and implementation of the system. There was also some knowledge of chemistry and biochemistry amongst the design team members.

The choice, and order, of activities (Figure 1) were based on: the nature of the data content, the availability of users, and the information we needed at the time. UCD approaches vary and this is just one manifestation of a UCD lifecycle. The dotted lines indicate the influence that each step had on the project; for example, user profiles (or ‘personae’, described later) were used throughout the project, such as for recruiting participants for workshops and usability testing. Each element shown in Figure 1 corresponds to specific pieces of work, so for a more detailed set of steps see the full workflow in the Additional file 1.

Ethical approval from an institutional ethics board is usually necessary when conducting usability studies with participants. Participant consent forms are often required and may vary depending on the institution and type of study conducted. In accordance with the EMBL ethics committee, all participants were asked to sign a consent form (see Additional file 2) and informed of their right to withdraw their participation at any time. Identifiable traces of participant data were removed and no data were made publicly available. All data handling conformed to the EMBL-EBI Terms and Conditions (http://www.ebi.ac.uk/about/terms-of-use).

We now give an account of each step in our UCD process.

### Stakeholder requirements were captured to define the scope of the project

We had an initial meeting with the project stakeholders, including UniProtKB [19], PDBe [20], ChEBI [21], Reactome [22], and research groups, who develop specialised databases such as MACiE [23] and CoFactor [24]. The two types of stakeholders were: (a) researchers who aim to raise the profile of their research output, and (b) service-providers, who already had their own service ‘brand’ and wanted to maintain visibility of that brand. The objective of the meeting was to capture the requirements and set expectations for the project. It was also explained that this process was going to adopt a user-centred approach.

This step ensured all stakeholders had an opportunity to contribute, and they understood the UCD process we would adopt for the project. We agreed to hold bi-weekly meetings to provide opportunities raise concerns.

### Personae (user profiles) were created to focus design for the users

An important tool in UCD is the ‘persona’. A persona is a user archetype, representing a major user group, that can help guide decisions about product features, interactions and navigation [25,26]. Their individual attributes, features and abilities are an amalgamation of data that is collected as part of user research carried out early in a project. Each persona can then be used, throughout the lifetime of a project, as a kind of lens through which to view elements and features of a website. For example, you can ask questions such as “How would removing feature X affect the workflow of persona Y?” This means that you can continue to keep the user at the centre of the design process. Furthermore, the persona is an important tool to allow empathy with the end user, as it reminds the development team that the user of the system has different goals to their own. In fact, Hudson [27] describes how the ‘self-as-user’ outlook in Baron-Cohen’s Empathising-Systemising Theory [28,29] may be mitigated by applying personae to a software design process.

**Figure 2:**
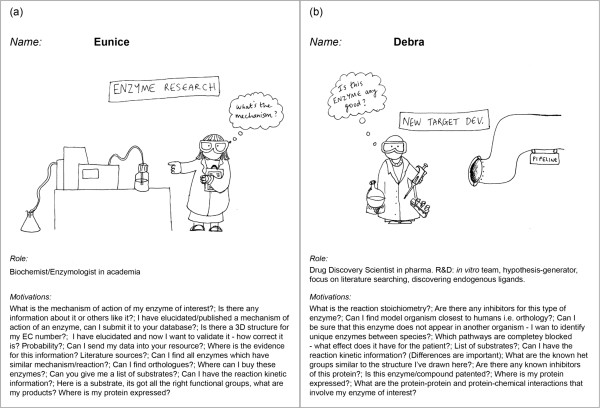
**Personae were developed by the stakeholders and refined by user interviews.** Two examples of the personae we created are (**a**) ‘Eunice’, and (**b**) ‘Debra’. Both represented bench scientists, with Eunice working in enzymology, and Debra in drug discovery. These personae, and three others shown in the Additional files 3 (‘Eric’ and and ‘Dean’) and 4 (‘Brenda’), reflected the expected desires and behaviours of potential users of the Enzyme Portal website. We described them in terms of name, role and motivations for using an enzyme resource.

We generated ideas for thirteen initial personae with input from the stakeholders. We decided to focus on just five of these, choosing those that had distinct motivations and behaviours, but all needing to access the Enzyme Portal data via the web interface, not via more advanced methods, such as programmatic access. The personae were given names, a short description of their job role, a set of research questions they might ask, and an image (Figure 2).

We focused on the ‘novice’ user for the first release of the Enzyme Portal. When developing a new bioinformatics service there is a tension between designing for the beginner (infrequent user) versus the expert (frequent user) [9]. This is because both bench scientists working in biological research (usually ‘novice’ users) and computational scientists working exclusively on the computer (usually ‘expert’/‘power’ bioinformaticians) may wish to use the same bioinformatics resource, albeit in different ways. For the Enzyme Portal, our sights were on the former, because we felt that the data to be presented would otherwise be very difficult to find and integrate manually, but could be easily achieved programmatically by a user with appropriate technical skills.

Indeed, it has been shown in the qualitative study reported by Javahery and co-workers [9] that novice users rated satisfaction with bioinformatics web interfaces lower - across several usability metrics - compared to expert bioinformaticians Hence, it can be harder to achieve a satisfactory level of usability for the novice. Accordingly, the decision to serve the infrequent user was reflected in the five personae chosen for the UCD of the Enzyme Portal.

### Interviews were carried out with users to validate the personae

Several members of the team had relevant experience in scientific research, hence our personae could be formulated on the basis of this experience, however we needed to confirm and refine the five personae by collecting information from the real people who represented them. To do this, we conducted one-to-one user research interviews, using the personae information to identify appropriate interviewees. For ‘Eunice’, Figure 2, (a), we interviewed a principal investigator at the University of Warwick. Likewise for ‘Debra’, Figure 2 (b), we conducted an interview with a senior scientist from a research-based pharmaceutical company. In this particular discussion, it became clear that the requirements of *in vitro* versus *in vivo* discovery scientists were distinct and should be separated; thus our original persona (not shown) was split into ‘Debra’ (*in vitro* discovery scientist, Figure 2 (b)) and ‘Dean’ (*in vivo* discovery scientist, see Additional file 3). The fifth persona -'Brenda', the biomarker research scientist - is in Additional file 4. The personae were edited after the interviews to reflect the ideas and language used by the interviewees [30].

Additionally, in the interviews, we confirmed the starting points and end points of a given user story, for example: would Eunice usually use a gene name, accession number or keyword to search the Enzyme Portal? What data would Debra need to feel satisfied after leaving the site? Essentially, we wanted to understand how the Enzyme Portal might fit into the context of a larger workflow, so we could construct plausible user journeys.

### Workflow analysis mapped out the behaviours and context of use for the personae

**Figure 3:**
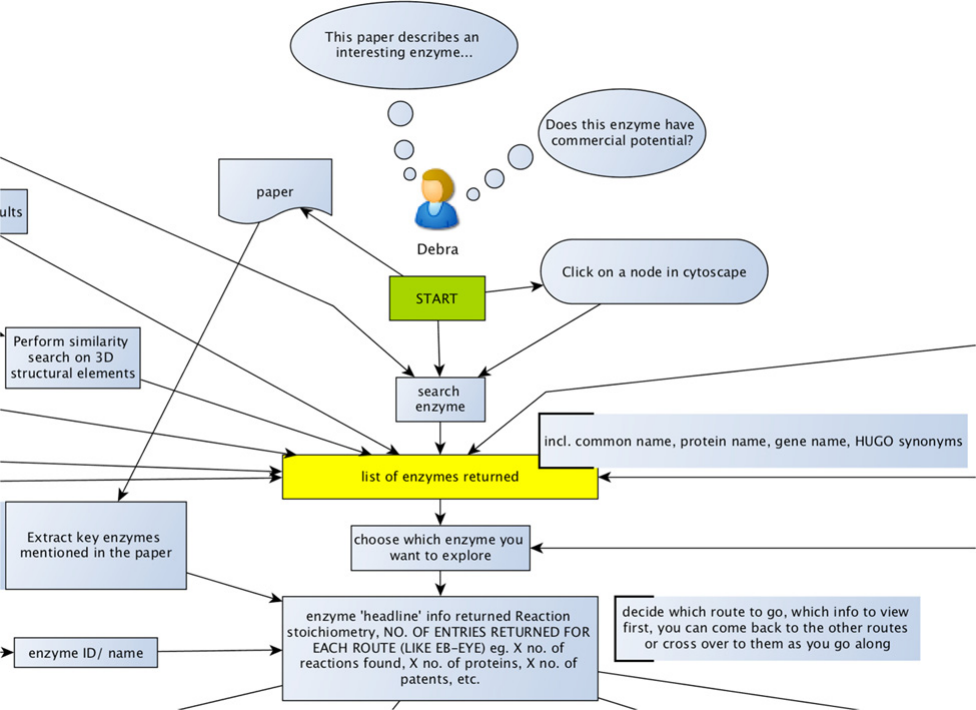
**Excerpt of the workflow analysis for the Enzyme Portal.** The workflow is a large and comprehensive model of how the user journeys for various personae interact with the Enzyme Portal, and serves to highlight commonalities in behaviour across personae. The thought bubbles indicate the motivation for the user to come to the Enzyme Portal, and the arrows indicate the flow through the layers of the site. The boxes represent individual webpages and list the data items expected to be displayed at each stage of the journey. The complete workflow is available in the Additional file 5.

To understand the flow of steps through the Enzyme Portal website, we created a map that included all the information from the personae and interviews in one diagram. We started by creating a formal task analysis diagram [31], with users on the left and the system on the right, and arrows denoting the information flow. But this rapidly became too complicated, and thus would be of limited value as a communication tool for the team or with users. Instead, we chose to create a workflow map (see Figure 3 for an excerpt, and Additional file 5 for the complete workflow analysis diagram).

The aim of workflow analysis was to identify key overlaps and ‘hubs’ in the Enzyme Portal site map. For example, it showed us that the headline/summary page was likely to be visited by virtually all personae, so this was an important page in the design. The major routes through the layers of the Enzyme Portal system were also identified in this way. Next, we refined the map by adding the findings from user workshops conducted with enzyme research experts.

Furthermore, when prototypes of the Enzyme Portal were ready (later in the process), the map served as a verification tool for testing the functionality of the site. For example, for usability testing sessions we could use the map to design relevant scenarios and tasks.

### Workshops with domain experts identified priorities for the design and provided information architecture

The output of our workflow analysis showed that when presented with the options, users wanted virtually *all* of the data we could possibly provide. Given the large amounts of data available in the databases for our project (UniprotKB, Reactome, PDBe, etc.), it was going to be a problem presenting all this data in a usable format; thus, we needed users to prioritise the information so it could be displayed within reasonably sized chunks that users could interpret. We also wanted users to categorise the information in meaningful ways: essentially to create a basic information architecture for enzyme biology and biochemistry. In light of this, we chose to conduct user-focused workshops where we would:

• provide a method for our users to *prioritise* the data and negotiate amongst themselves the importance attached to each type of data, ultimately reaching consensus.

• facilitate a discussion on how they wanted to *interact* with the data, and how it would fit with their existing research tasks.

• *confirm and refine* the information we had collated from user interviews, and the user workflows. For example, one participant suggested an entry point to the Enzyme Portal should be via a scientific paper, not just via an enzyme name - this was an addition to the workflow for the ‘Eunice’ persona.

We carried out two workshops: one at EMBL-EBI with ten experts from academia and a pharmaceutical research and development (R&D) organisation; and another at an agrichemical company R&D site with six plant researchers and bioinformaticians. The participants were recruited based on their similarity to the personae, and included principal investigators, researchers and PhD students.

**Figure 4:**
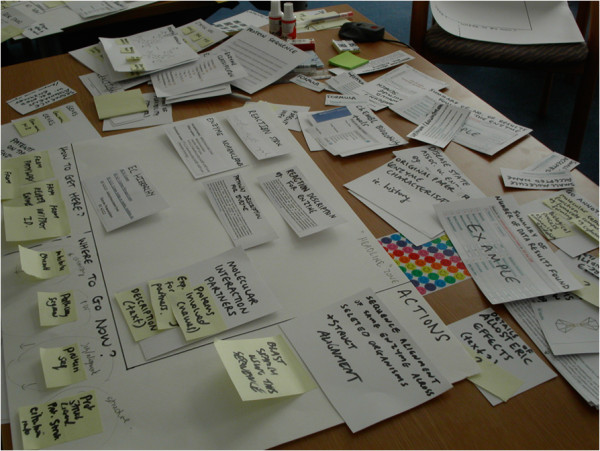
**Photograph of the materials used in the canvas sort activity with user workshop participants.** A novel ‘canvas sort’ approach was used in workshops with users to elicit information architecture, and to prioritise enzyme-related data items.

We prepared activities to elicit feedback from the participants. For example, to address the issue of data prioritisation we came up with a new variant of a card sorting exercise called, ‘canvas sort’ (Figure 4). The standard way to perform card sorting for web design involves participants putting item cards, which represent information on a website, into separate piles and giving each pile a group name. In an open sort they write their own names, whereas in a closed sort they use pre-labelled group cards [18]. This method gives a picture of the users’ mental model of the items.

In contrast, our method required that users select the most valuable data items for their research, and arrange them in a structured fashion on canvas template, see Table 1. The data items are listed in the Additional file 6; they included data items such as “enzyme reaction”, “enzyme mechanism” and “chemical compound/ligand”. The specific components users had to place onto the canvas included data items (nouns) and interactive elements (verbs), see Table 2. We carried out a pilot study with the project stakeholders to ensure the canvas sort method was effective before using it in the workshops.

**Table 1:** The structure of the canvas used in the canvas sort activity during user workshops

**Canvas name**	**Actions**
	
** Data card selection**	
	
How to get here?	Where to go next?
	

The canvas sort activity was divided into four main sections one for data cards and the other sections were used to describe the interaction on those data cards.

**Table 2:** Summary of the components of the canvas sort activity

**Component added by the users to the canvas**	**Description of the component**	**UCD information and useful outputs from the component**
Canvas name	Users were required to select the name of the canvas and to choose data cards to put onto the canvas.	Category names, in the users own words, were useful for the site map, information architecture and for understanding the overarching concepts that were important to the users.
Data card selection	Data cards were based on data items extracted from our existing resources and also requests during the interview process such as “enzyme reaction”, “enzyme mechanism” and “chemical compound/ligand”. These were printed in duplicate onto white card and cut out to any reasonable size. Blank cards were also available so users could create their own data items if none of the available were appropriate. After selecting a number of data items users had to democratically vote to keep only six using the dot voting method [as described in [29]).	This was the prioritisation task of the exercise. By allowing only 6 items to be collated on one canvas, it forced participants to prioritise which were most valuable to them.
		An incidental benefit of this approach was that users’ awareness was raised, regarding the data items we could provide via EMBL-EBI public data resources.
Actions	On sticky notes users had to describe what actions they wanted to perform on these data items.	Nouns (data items) and verbs (actions) needed to be captured in the workshop in order to translate them into specifications for the Enzyme Portal design.
How to get there?	Describe how they could navigate to this canvas.	This gave insight into the navigational structure that users required.
Where to go next?	Describe where they would want to get after this canvas.	This gave insight into the navigational structure that users were after.

The main purpose of the canvas was to identify what data was valuable to users at each point of the workflow and how users wanted to interact with the data. The workflow was also captured by the final two sections, which describe how users got to this canvas and where they would like to go next.

Canvas sorting was carried out in groups of three or four participants. We avoided power relationships in a group, for example, we did not put PhD students with their supervisors, and where possible, we avoided grouping people from the same organisation together. We also had a mix of user profiles represented in each group to try and achieve consensus.

The agenda included an ‘ice breaker’ activity to increase familiarity between the members of each team. The rules of the canvas sort activity were explained using a non-scientific example, in this case a retail website for books. The activity was split into three parts: 40 minutes for the activity, ten minutes for each team to report back their findings, and a few minutes for discussion with the other teams; thus it followed Gray *et al.*’s suggested ‘opening’ (setting the rules), ‘exploring the world’ (playing the game) and ‘closing’ (presenting back) of creative ‘game-storming’ techniques for team-based workshops [32]. We video-recorded the reporting back sections. Each group also had a facilitator to stimulate and record discussion amongst the team members.

### Analysis of the workshop findings informed design decisions

**Figure 5:**
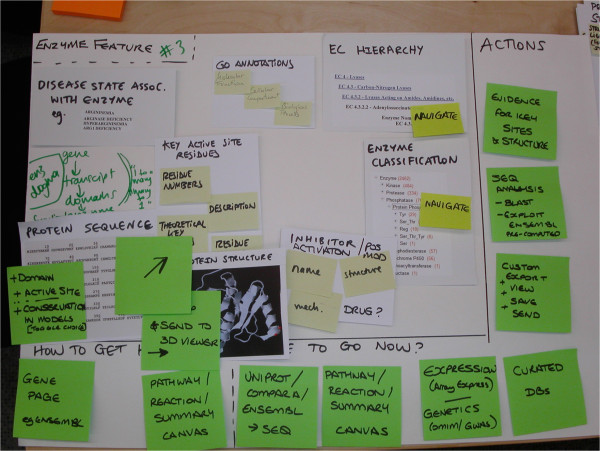
**Photograph of a completed artefact generated in the canvas sort activity by user workshop participants.** These artefacts were useful for prioritisation of data items and functionalities when planning the structure of the Enzyme Portal. There were five teams and each team produced 3 or 4 of these in the workshops**.**

After the workshops, we photographed the canvasses; see Figure 5 for an example. Using these artefacts and the videos, we manually noted the specific data items, actions and navigation for each canvas and grouped canvasses with similar-sounding names across groups (Table 3). For designing the Enzyme Portal interfaces, we prioritised data items, functionality and navigation that co-occurred in these findings.

**Table 3:** A portion of the matrix of commonalities for the reaction canvas in the canvas sort user workshop activity

**Data items**	**Group 1**	**Group 2**	**Group 3**
Overall reaction	✓		
Postulated mechanisms	✓		
Evidence of mechanism	✓		
Kinetic constants for various steps in the mechanism (organism and substrate specific)	✓	✓	✓
Reaction in pathway			✓
Enzyme inhibitors / activators			✓
Literature information i.e. original paper and most recent work on the enzyme			✓
Bioactivity data		✓	
Chemical group information (classification)		✓	
Chemical compound information (structure, formula, etc.)		✓	
Compound supplier information		✓	
Safety information on this compound		✓	

This canvas illustrates the commonalities of the selection of data items by workshop participants. In this case only three groups agreed on a reaction canvas.

**Figure 6:**
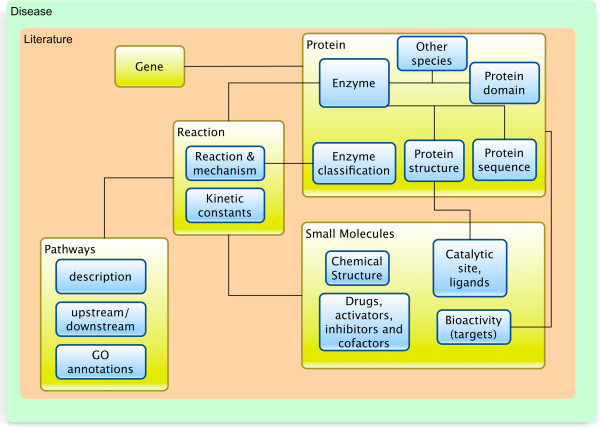
**Concept diagram of the information architecture applied in the Enzyme Portal.** The data objects are grouped and each category has a number of data items valuable to the user. Categories and data objects are then interconnected. Concepts that did not fit into this model were disease and literature, which span across all the data objects and categories.

The canvas names and details formed the backbone of the design for the website’s navigation. Using the findings, we were able to abstract an information architecture for enzyme biology relevant for the Enzyme Portal (Figure 6). It illustrates the major connections between protein, reaction, pathways and small molecules from the users’ perspective.

### Paper prototypes made it easy to share ideas for the design

We created paper prototypes (static images) for the Enzyme Portal and used the information from earlier user research, such as the interviews and workshops, to guide the visual designs. Paper prototypes are simple representations of a user interface in different states, and allow rapid, iterative design of pages and their transitions [33]. Often they are simply sketches or printouts of interfaces that are used in the early stages of development to try out ideas quickly [34,35]. Although they lack the complete functionality, sketches can be used to test users’ interactions with a proposed interface as they contain real data examples.

We ensured that the functionalities requested by users were included in our prototype designs. We used paper and pencil to sketch ideas individually and we discussed each design idea as a group. Good ideas were incorporated into a set of paper prototypes using the Balsamiq (http://www.balsamiq.com) toolkit, see Additional file 7.

### Paper prototype usability testing highlighted problems with the design

Paper prototype testing is beneficial because the overhead of design is low, since time is not invested producing high-fidelity prototypes. When testing them with users, participants are free to concentrate on exploring features, without the distraction of high-fidelity designs, interactions and colours. We could also test them with users at a very early stage. Moreover, because the prototypes were on paper, they could be rapidly redrawn and redesigned when usability issues emerged during testing.

**Figure 7:**
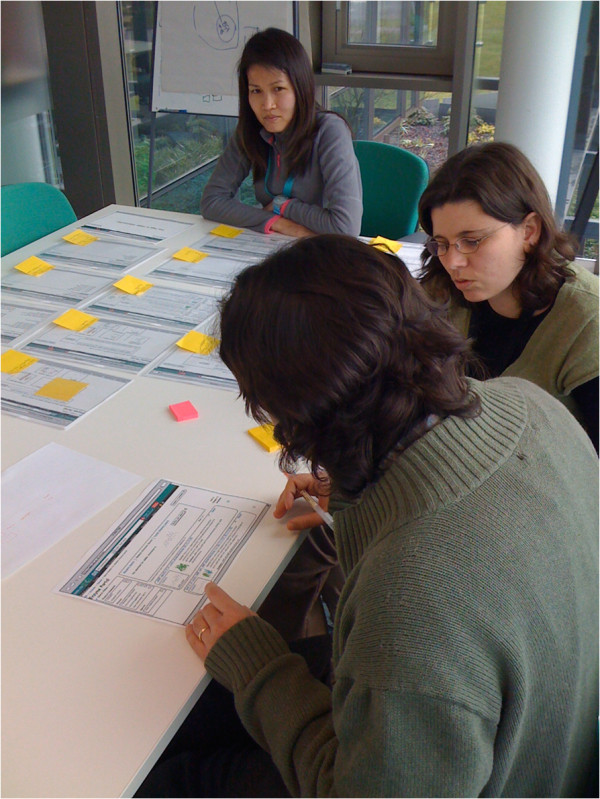
**The paper prototyping method was used for usability testing of initial designs for the Enzyme Portal.** The user was presented with a paper wireframe of each screen interface and could interact with it using a pencil instead of a mouse and keyboard. Each screen would then change according to the interaction. The wireframe interface designs were created using Balsamiq software (http://balsamiq.com).

In a paper prototyping experiment, test participants are briefed about what to expect in the testing, and its purpose, then they are asked to complete tasks using prototype interfaces on paper, and are requested to talk out loud while they do this [33]. If a participant clicks on a button with their pen, for example, then the paper prototype they see is exchanged for another that displays what would be shown on screen as a result (Figure 7).

We observed participants’ reactions to the proposed features and for transitions from one state of the interface to another; for example we could observe users checking the faceted search results to filter the results set. We chose cGMP-specific 3’,5’-cyclic phosphodiesterase (PDE5A) enzyme and its inhibitor, the drug Sildenafil, as the example data for the testing sessions and for the scenario and tasks for user testing (see Additional file 8). We chose PDE5A because: a) this enzyme was well characterised having data available across several biological and chemical databases that we were integrating in the Enzyme Portal, and b) it was cited as a relevant example by a user in the interviews conducted earlier in the process.

We carried out testing with four participants, who were recruited via email to a UK University. We felt that this relatively small number was acceptable, since only five or six users per evaluation results in most usability issues being identified [36]. Participants had a variety of backgrounds including medicine and computer science. Our aim was to determine if the flow of steps through the service seemed intuitive for a new user. The test involved: a moderator, who prompted the user; an observer, who took notes; and a developer, who passed the papers representing screens to the moderator. A rehearsal was performed to ensure that the task we had designed would elicit suitable interactivity in the time available. We used a quiet room, obtained consent, and gave participants opportunity to talk about their own research to put them at ease. We explained that our goal was not to test their skills, but rather the capabilities of the interface.

Directly after the sessions, we collated our findings and ordered them by priority, based on the number of times a usability problem was observed. We analysed the video footage, and used it as a communication tool with the team. Some design problems became apparent immediately and could be solved easily, such as the size of the main search box on the homepage. But others were harder to fix, such as the elements to display in the post-search results list.

### A technical specification was written for software development

The specification document included visual design layouts based on the prototypes we had refined post user-testing. The advantage of having detailed specifications at this point was that there was less ambiguity, so it was easier for the software development team to follow and produce a system that faithfully reflected the requirements from users.

With well-tested specifications the team could carry out the implementation without interruptions due to requirement changes, which can often occur with other methods of software development. Moreover, the developers could focus on the main technical challenges of data integration, such as where performance had to be considered when data is retrieved from disparate databases via queries to different Web service interfaces. Several technical solutions were discussed among the stakeholders to overcome these type of challenges. Performance issues were solved by applying appropriate methods of querying the data, which were suggested by the data providers themselves. Furthermore, performance was enhanced by applying a concurrent programming technique, where several queries are performed simultaneously. The adapter design pattern was applied to combine data from several interfaces into a single interface to facilitate the queries. The implementation was concluded successfully, with the first version of the interactive prototype released for testing. It is a Web application built on the Java platform (http://www.oracle.com) and Web technologies such as Spring MVC (http://www.springsource.org/), jQuery UI (http://jqueryui.com), HTML and CSS (http://www.w3.org). External interfaces from which data is queried include Distributed Annotation System (DAS) [37], BioMart (http://www.biomart.org/) and the data providers’ propriety interfaces.

### Designs were further refined after usability testing of an interactive prototype

For usability testing of the interactive prototype, we recruited five participants from R&D organisations in the pharmaceutical, chemical and agri-chemical industries. The interactive prototype was tested using MAC OS and a Windows 7 platform, depending on participant preference. An observer and moderator were present and the sessions were screen recorded using Silverback Software (http://silverbackapp.com/) and BB Flashback Express (http://www.bbsoftware.co.uk/BBFlashBackExpress/Home.aspx), respectively. Consent was obtained.

**Figure 8:**
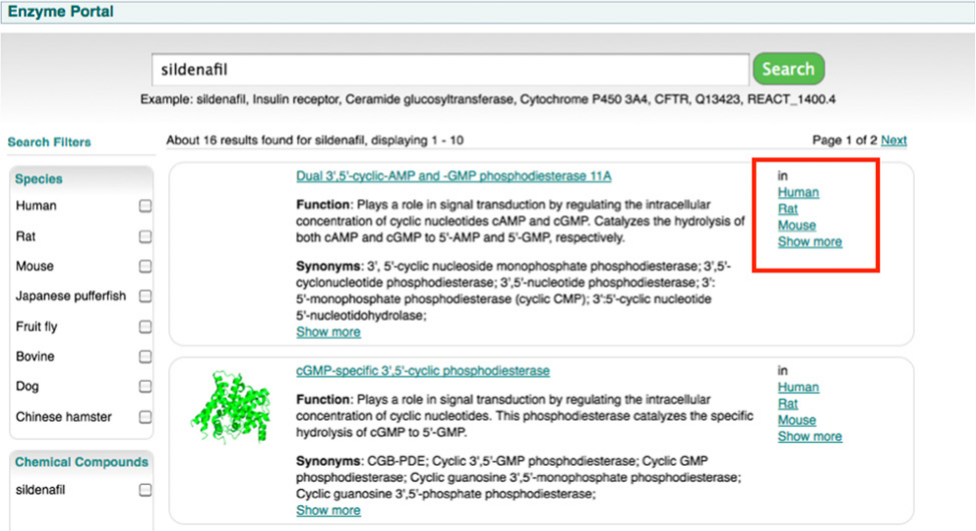
**The first version of the search results page using an interactive prototype.** In this version not all the protein structures were displayed and also the species were located on the right hand side of each entry found.

**Figure 9:**
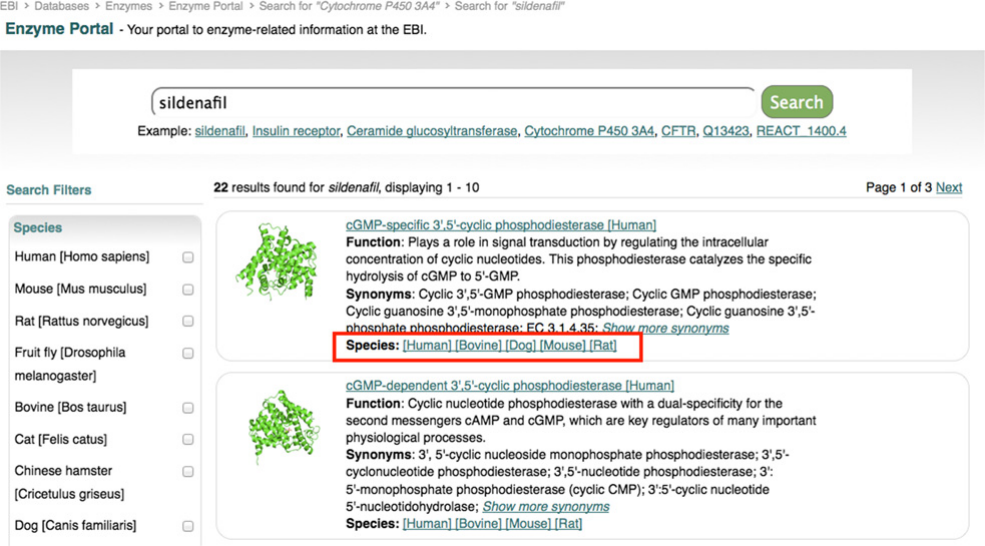
**A later version of the search results page after improvements highlighted during interactive usability testing.** In this version the species are located below the synonyms, and protein structures is displayed for an entry irrespective of the default species.

We summarised the key findings and discussed them with stakeholders. The system was refined based on these findings; for example: the location of the list of species available for each enzyme ‘hit’ was rearranged, because users could not locate them. Figure 8 illustrates the first interactive prototype, which had the species on the search results page on the right-hand side of the box in the search results hit list (highlighted in red). Usability testing highlighted that users did not see this information on the left-hand side either. The solution was to move the species list below the function and synonyms section (Figure 9) (highlighted in red).

## Results

Historically, enzyme information at the European Bioinformatics Institute website was organised across multiple data resources, which were loosely hyperlinked. Initial interviews indicated that users struggled to identify which resources were available, and could not navigate the information, as it was not logically grouped together and was not easily searchable. Users also pointed out that critical information was missing (e.g. disease). The Enzyme Portal addresses these issues in a single system, whereby disparate resources, including a protein knowledgebase [19], various other biological and chemical databases [20-24,38,39], experimental factor ontology (EFO) [40] and literature citations (http://www.ebi.ac.uk/citexplore), may all be explored in a single visual display after a single search.

### Summary of insights uncovered through UCD

Specific user expectations were revealed as a direct result of applying UCD techniques to develop the Enzyme Portal. We do not report all of the learnings we gained from interaction with users here, however a summary of key findings that inspired the design is below. Note that these observations were applicable to the personae we were designing for.

• Users do not want enzymes grouped by EC number alone, they also require an ontology that fits with a pharmacologically-relevant schema.

• Users want entries to be species-specific, not an average combined model of a structure across the protein found in several species.

• Users want to compare the list of characteristics of two or more enzymes on the same page, as one might compare specifications of retail products side-by-side on a commercial website.

• The enzyme function is really important and is a key decision maker on whether to take a deeper look at the enzyme.

• Users want the information embedded not hyperlinked, to save time clicking to and from multiple webpages/ browser tabs.

• Protein structure is essential information when looking at an enzyme. Thus, presenting thumbnails to indicate whether a structure is available is handy for determining which enzymes to explore further.

• All enzyme synonyms should be searched on, not only those approved by the NC-IUBMB. This is because enzymes may have been referred to by different names in the literature, so the scope of the search must include these alternatives

• Users want to forward information to colleagues easily and also download time-stamped data for further reference.

• Facets are desirable for narrowing down the search results based on the user’s criteria, such as species.

• Information about disease is under represented in most publicly-available databases. Users want this information categorised separately.

• In the canvas sort activity, users categorised information in the following key categories: small molecules, reactions, pathways, protein structure and enzyme summary.

### Enzyme portal is a freely-available service hosted at EMBL-EBI

The Enzyme Portal was released in February 2012 (http://www.ebi.ac.uk/enzymeportal), with subsequent releases planned for the short term to address outstanding functionalities identified in our UCD process. Interactive prototype testing conducted a month prior to release indicated that users liked the features the Enzyme Portal has delivered. The layout and categorisation of the data has also been well received. As mentioned, minor issues were uncovered in navigation, such as how the available species information was displayed for each search hit (as shown in Figure 8 and 9), and these were fixed for the final release.

There are still outstanding technical and data issues that prevent the Enzyme Portal from delivering all aspects of the user requirements we discovered. For example, we did not include the side-by-side enzyme comparison tool, and the species search facets in a separate pop up window, in the first release. We intend to resolve these omissions in subsequent versions of the Enzyme Portal. Further user testing has not been conducted since the release, and although the portal is in its infancy informal interviews with users who have been introduced to the portal through the EBI’s training programme have heralded it a success.

We see UCD as an overarching philosophy, and thus we plan to continue monitoring user interactions with our new service going forward; for example, through website usage analysis and continued usability testing of software updates.

## Discussion

### Did the UCD approach work?

We have applied UCD thinking to the design and development of a new bioinformatics service called the Enzyme Portal. Many new bioinformatics services and data integration portals are described in the literature, such as those published each year in the database issue of Nucleic Acids Research journal [41], however few have undertaken a UCD approach to develop them as we present here. Furthermore, we believe there is a need to increase visibility of UCD approaches in the community at large, as Veretnik *et al.* suggest: “*The issue of persistence and usability…plays a significant role in how our discipline is perceived*” and “*there are scientists themselves who publish the work but do not want to go to the trouble of making the resources easy to use…Wouldn’t it seem that evidence of usability…should be prerequisite to publishing a paper …about such a resource?*” [42].

Our motivation for applying UCD to the Enzyme Portal was to create a service based on the expectations of our users, rather than on our underlying data structure and our own assumptions of what users would want. We have shown how a UCD philosophy may be applied in bioinformatics and provide materials for general use by developers of bioinformatics applications, such as a card sorting template, an example of a consent form, etc.. In summary, we presented the practical steps involved to realistically achieve improvements in usability: from stakeholder analysis, user research and persona development to prototyping and usability testing.

We found that the UCD process is more complex than the traditional ‘waterfall’ software development cycle [43] applied to the development of most new bioinformatics services. However it offered distinct advantages to the traditional methods of development. For example, the decision-making process was simpler at each stage of the design, because we had clear requirements from users to follow. Furthermore, using this approach we had physical artefacts from workshops and visual sketches as communication tools to explain what was needed within the team. These were helpful for preparing both visual and functional specifications for implementing the software.

Other benefits of the UCD methodology included access for the development team to feedback from users immediately after each section was implemented, rather than receiving comments after all development was completed, when the site could not be easily changed; as often happens in bioinformatics software development. Another real, yet intangible, benefit was the opportunity that UCD provided for enhanced team-working, where developers, stakeholders, user experience practitioners and project coordinators were all practically involved, including for pre-implementation activities, such as persona development, user testing and sketching designs.

Most importantly the UCD process prevented us from pursuing false avenues and challenged our assumptions about the data we were integrating and displaying: for example, in our decision not to focus on enzyme commission (EC) numbers [44], but rather on other hierarchies, such as ChEMBL enzyme bioactivity classification. Overall, we believe it allowed us to deliver a better experience for the end user, compared to using a standard waterfall software development process.

There were some limitations to the UCD process we applied; for example, software development was initially delayed while we were characterising the needs of our users, hence no measurable output could be provided up-front. However, once development did start, developers reported that they enjoyed coding the project, and felt they were coding efficiently because they had a clearer visual specification to work from.

Initially, there were problems in convincing the team, stakeholders and management of the benefits in adopting the UCD process. This was primarily because there was an initial cost for gathering requirements, but in the long term this was negated by the faster development time.

Demonstrating the advantages of UCD was challenging, because the benefits are only realised after the software is released, and even then, they may be intangible - such as impacting on early-stage scientific discoveries and basic research – thus are hard to quantify and communicate to stakeholders. However, the intangible nature of the ‘value’ of such services is the same for most bioinformatics services.

## Conclusions

We hope that the approach used to design the Enzyme Portal will motivate the reader to apply UCD to their own bioinformatics tools and services, because we believe that if you design a product in consultation with the user, you will likely find that more people will use it and will benefit from your offering - even if these benefits may be hard to measure. For the bioinformatics community, getting the design right is key, in order that the scientific community may reap the benefits of the public spend to create data from scientific endeavour. It is our duty to make the data available so scientists can find and manipulate it easily. We believe UCD may provide a way to achieve this ambition in the future.

## Abbreviations

EC: Enzyme Commission; EMBL-EBI: European Molecular Biology Laboratory - European Bioinformatics Institute; PDE5A: cGMP-specific 3’,5’-cyclic phosphodiesterase; UCD: User-centred design

## Competing interests

The authors declare that they have no competing interests

## Authors’ contributions

PdM and JC planned and carried out the UCD work described, and wrote the manuscript. HC and RA were responsible for the software development for the Enzyme Portal. FR provided advice and participated in UCD and testing sessions. RL was responsible for supporting the technical infrastructure for the Enzyme Portal. CS oversaw the project. All authors edited the article. All authors read and approved the final manuscript.

## Supplementary Material

Additional file 1 Complete user-centred design (UCD) workflow for the Enzyme Portal. Click here for file

Additional file 2 Usability study participant consent form. Click here for file

Additional file 3 Personae ‘Eric’ and ‘Dean’. Click here for file

Additional file 4 Persona ‘Brenda’. Click here for file

Additional file 5 Complete task flow diagram for the Enzyme Portal. Click here for file

Additional file 6 List of data items used in the Canvas Sort. Click here for file

Additional file 7 Balsamiq paper prototypes for usability testing of the Enzyme Portal. Click here for file

Additional file 8 Enzyme portal prototype testing, scenario and tasks. Click here for file
