# Supplementary figures and images for: The Enzyme Portal: a case study in applying user-centred design methods in bioinformatics
Source: BMC Bioinformatics. 2013 Mar 20;14:103. doi: 10.1186/1471-2105-14-103 (PMC3623738; doi:10.1186/1471-2105-14-103)

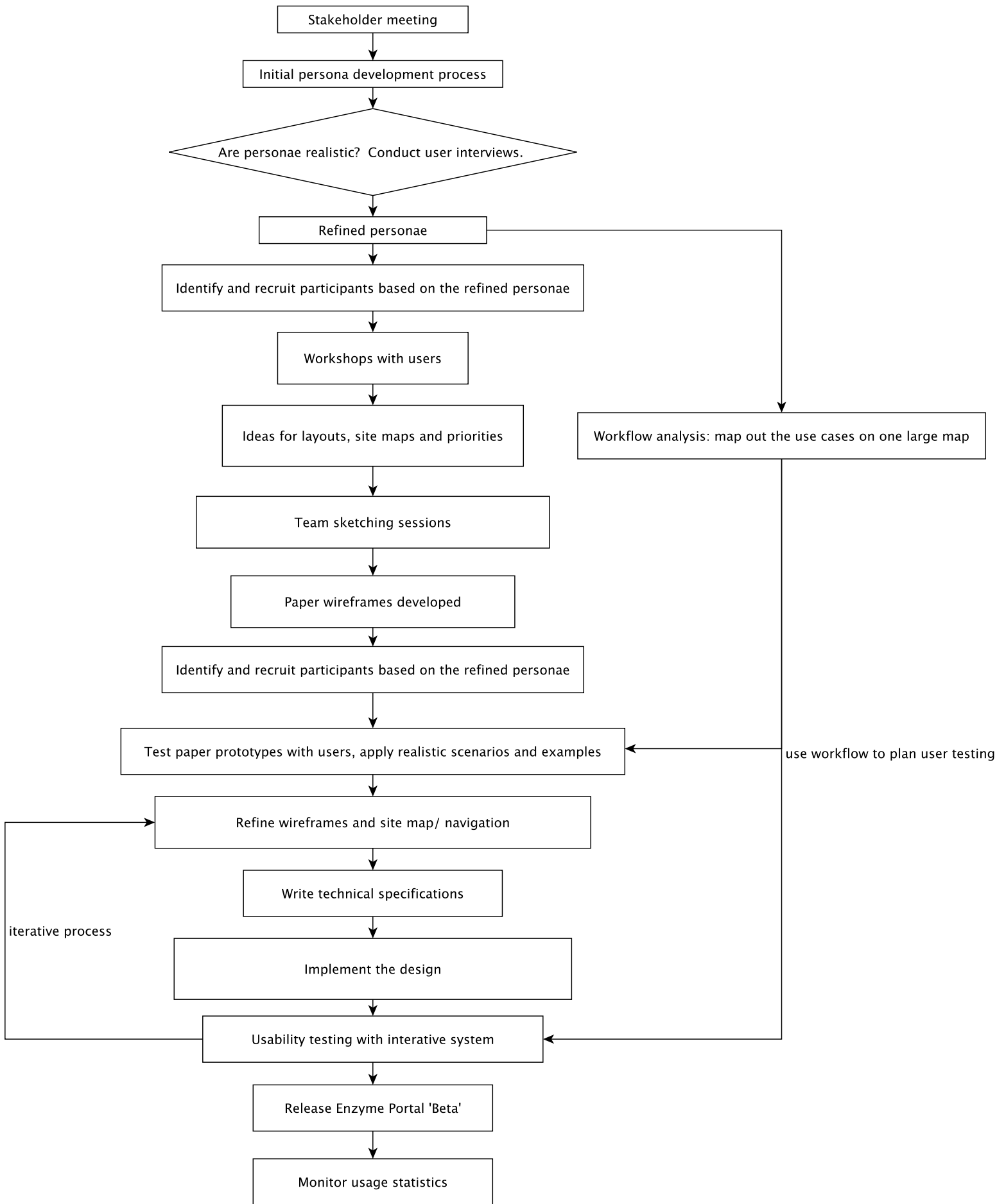

Supplement: Additional file 1 — Complete user-centred design (UCD) workflow for the Enzyme Portal. [file 1471-2105-14-103-S1.pdf]

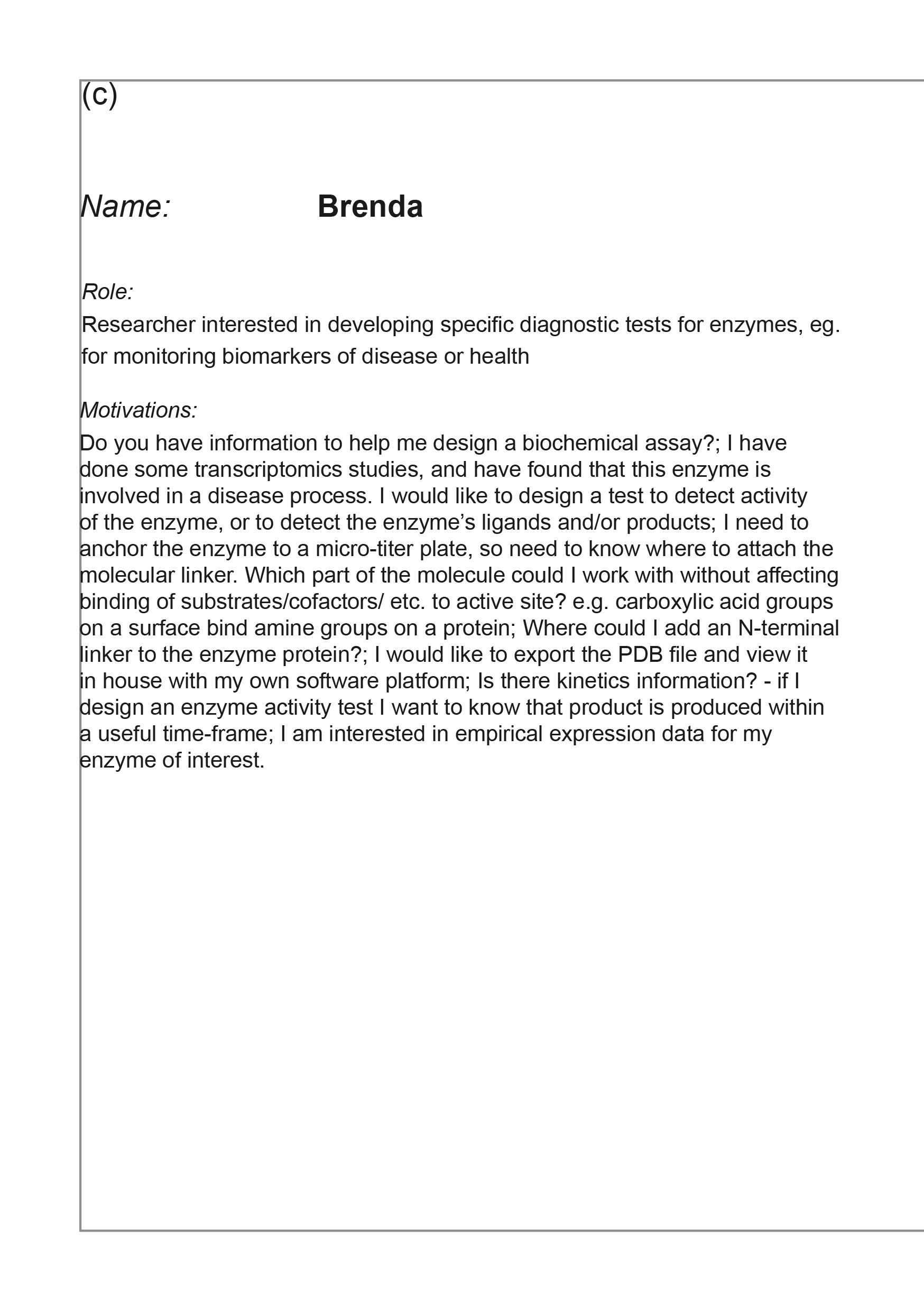

Supplement: Additional file 4 — Persona ‘Brenda’. [file 1471-2105-14-103-S4.tiff]

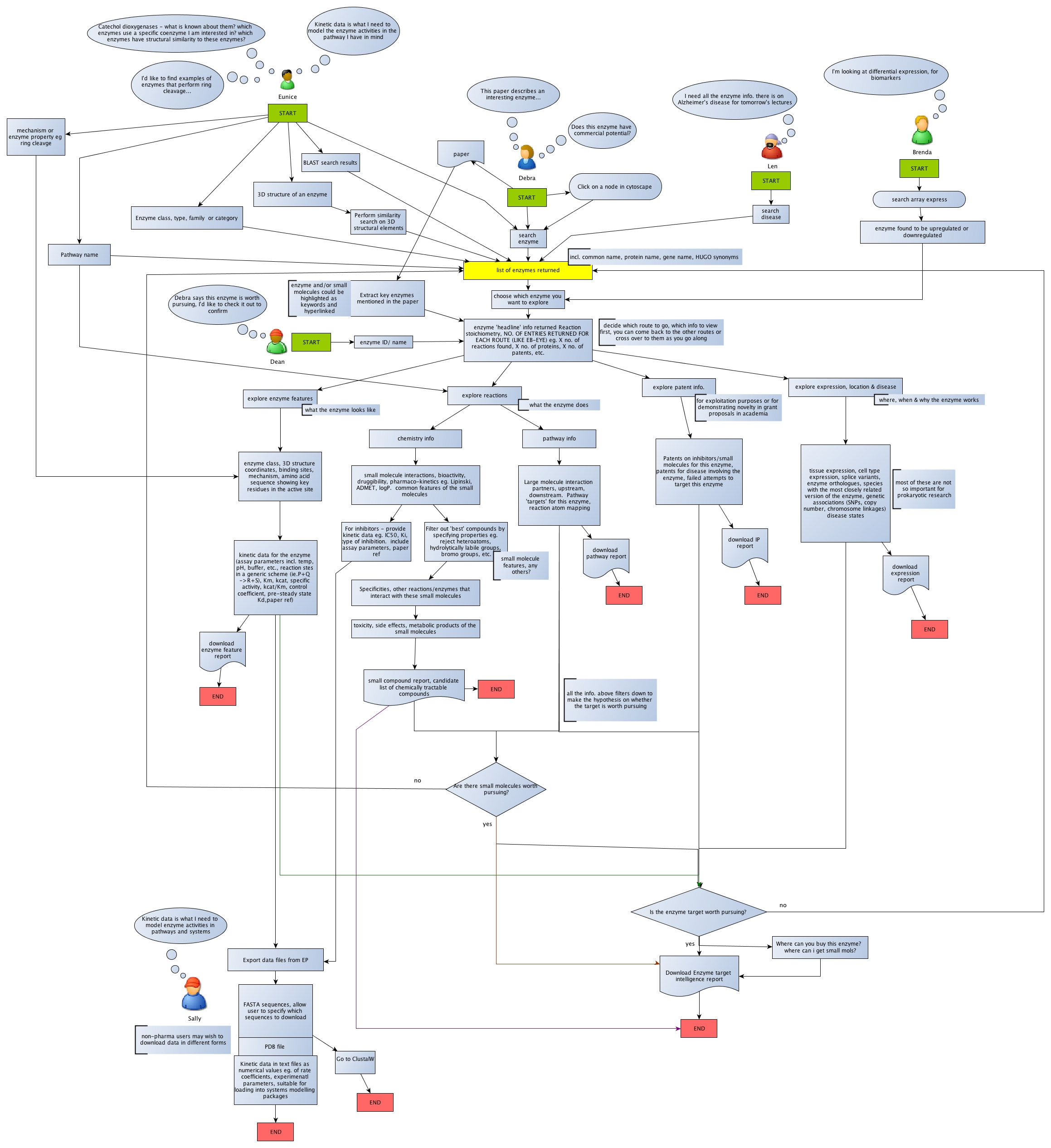

Supplement: Additional file 5 — Complete task flow diagram for the Enzyme Portal. [file 1471-2105-14-103-S5.tiff]

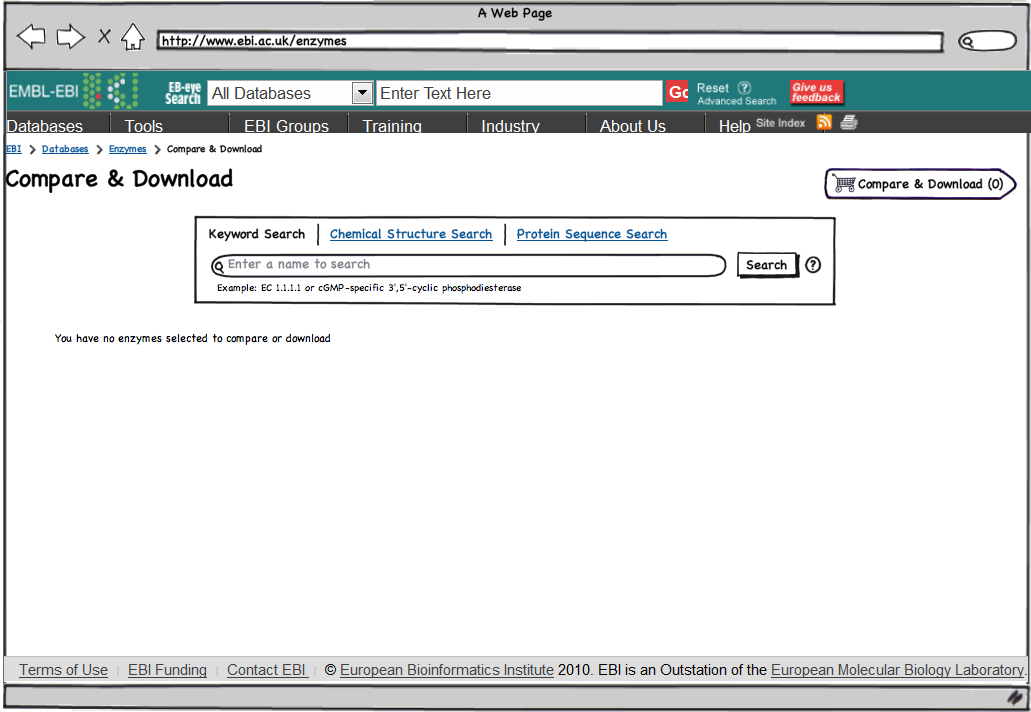

Supplement: Additional file 7 — Balsamiq paper prototypes for usability testing of the Enzyme Portal. [file 1471-2105-14-103-S7.zip › paper prototypes/Basket View None.png]

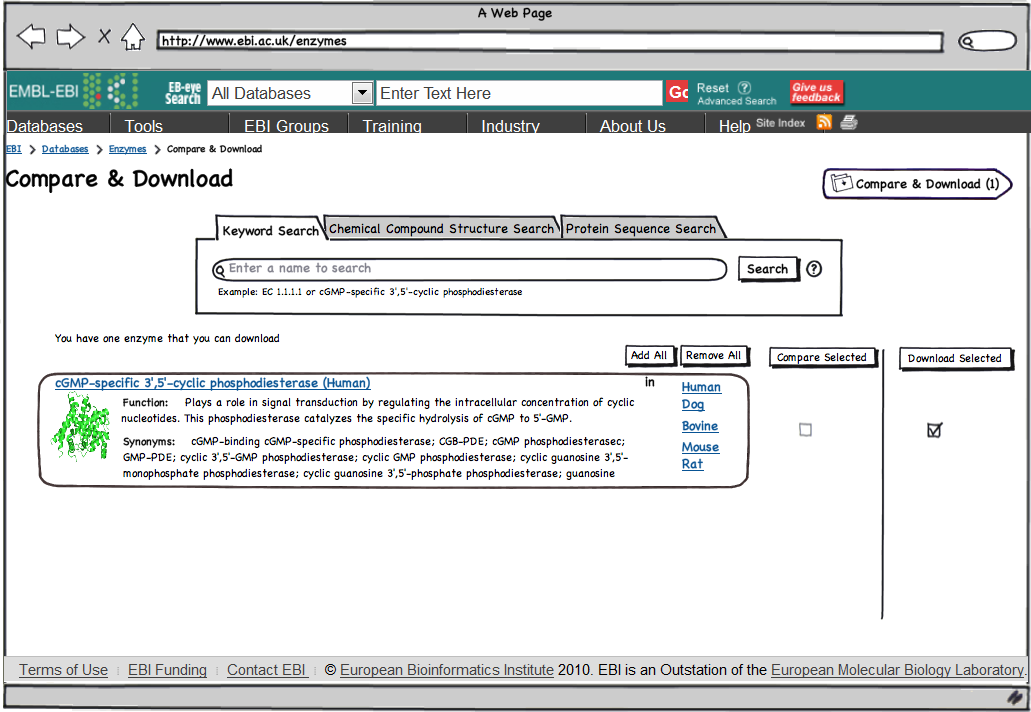

Supplement: Additional file 7 — Balsamiq paper prototypes for usability testing of the Enzyme Portal. [file 1471-2105-14-103-S7.zip › paper prototypes/Basket View Only one.png]

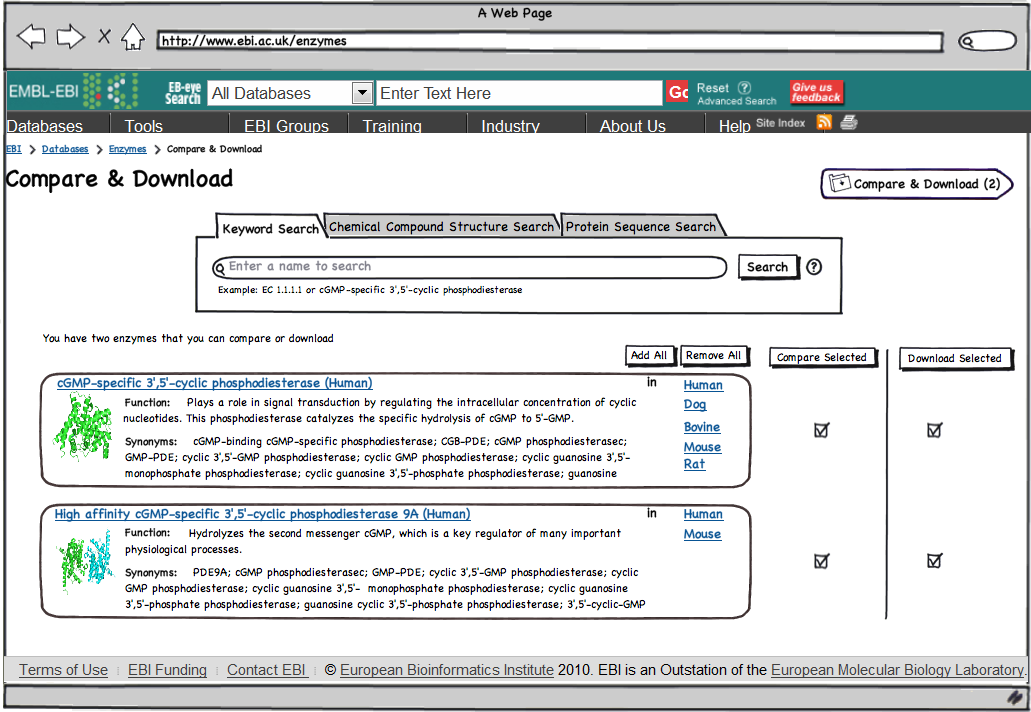

Supplement: Additional file 7 — Balsamiq paper prototypes for usability testing of the Enzyme Portal. [file 1471-2105-14-103-S7.zip › paper prototypes/Basket View.png]

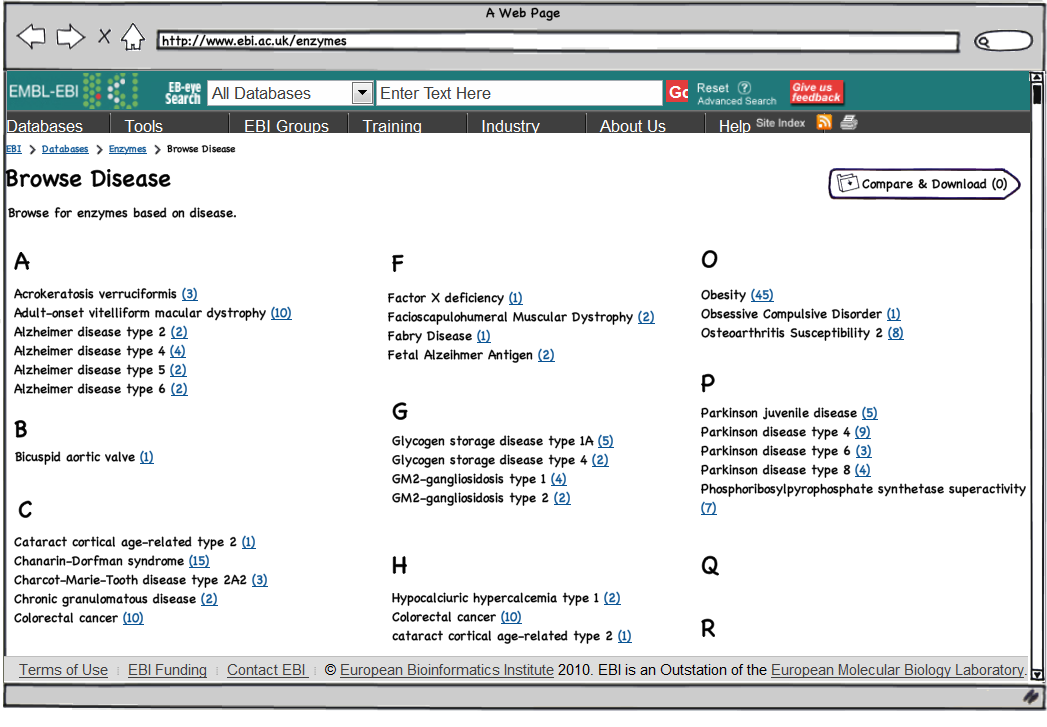

Supplement: Additional file 7 — Balsamiq paper prototypes for usability testing of the Enzyme Portal. [file 1471-2105-14-103-S7.zip › paper prototypes/Browse Disease.png]

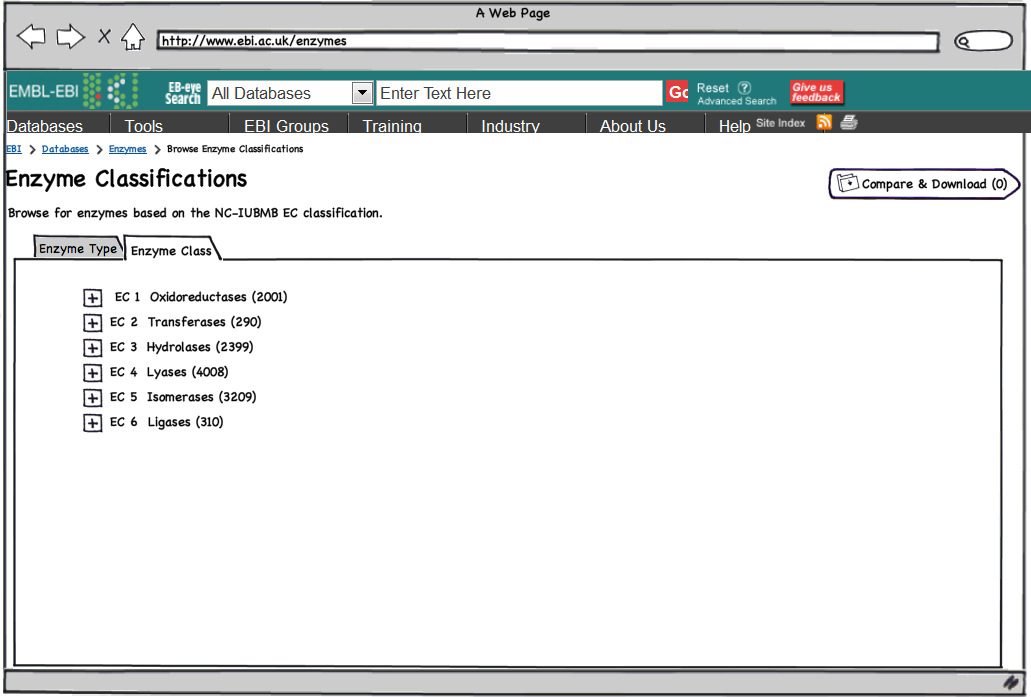

Supplement: Additional file 7 — Balsamiq paper prototypes for usability testing of the Enzyme Portal. [file 1471-2105-14-103-S7.zip › paper prototypes/Browse EC Classification.png]

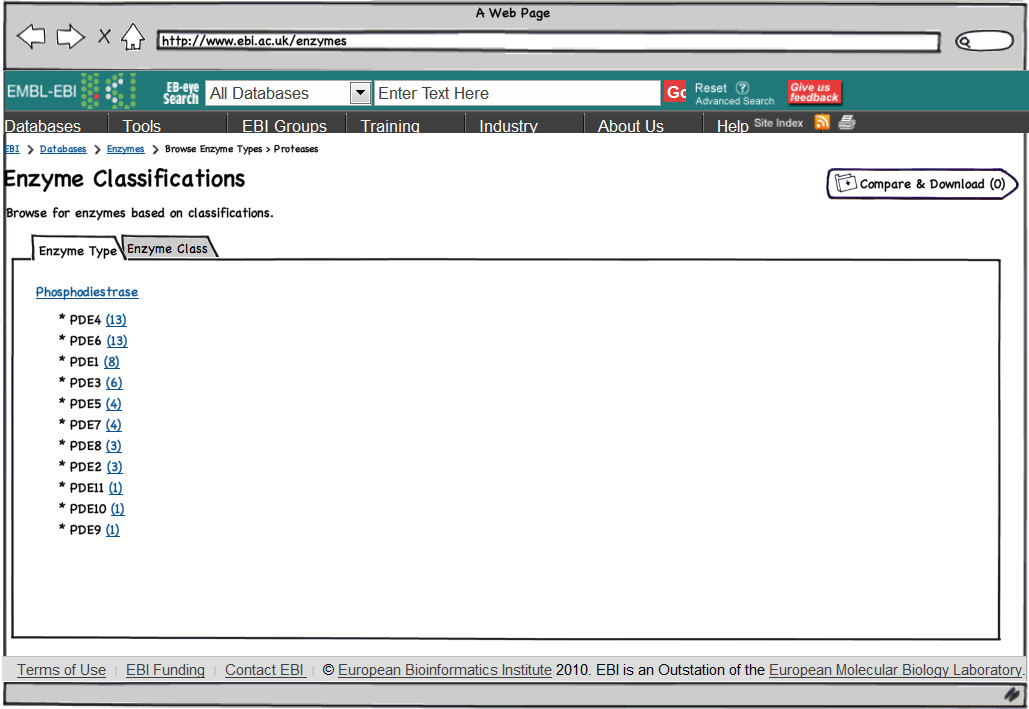

Supplement: Additional file 7 — Balsamiq paper prototypes for usability testing of the Enzyme Portal. [file 1471-2105-14-103-S7.zip › paper prototypes/Browse Enzyme by Type Page 2.png]

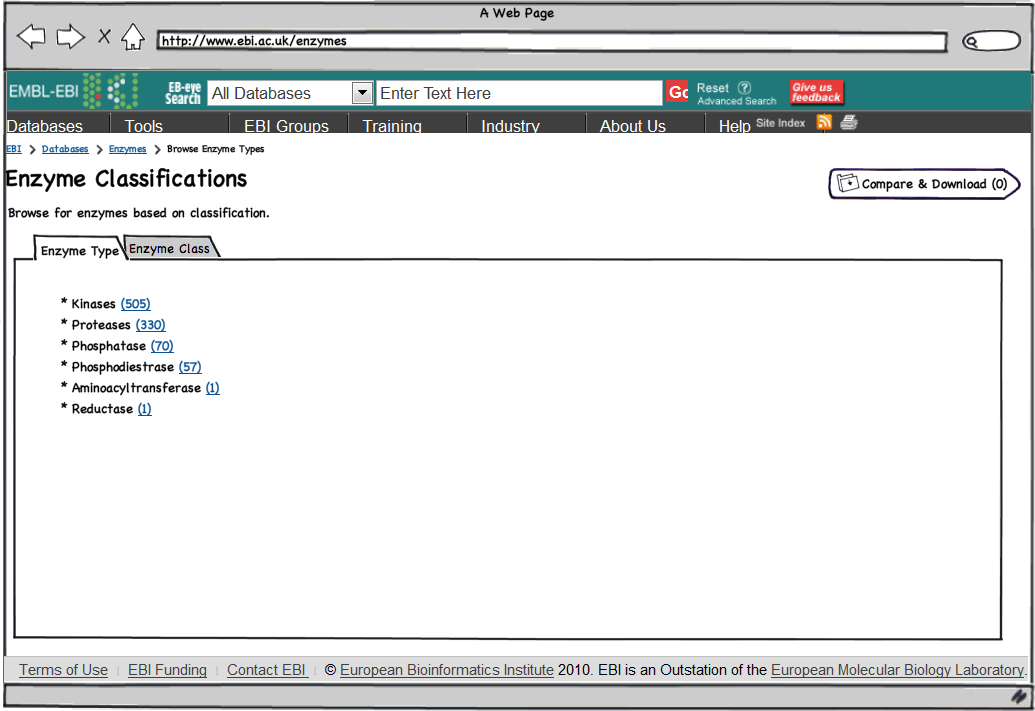

Supplement: Additional file 7 — Balsamiq paper prototypes for usability testing of the Enzyme Portal. [file 1471-2105-14-103-S7.zip › paper prototypes/Browse Enzyme by Type.png]

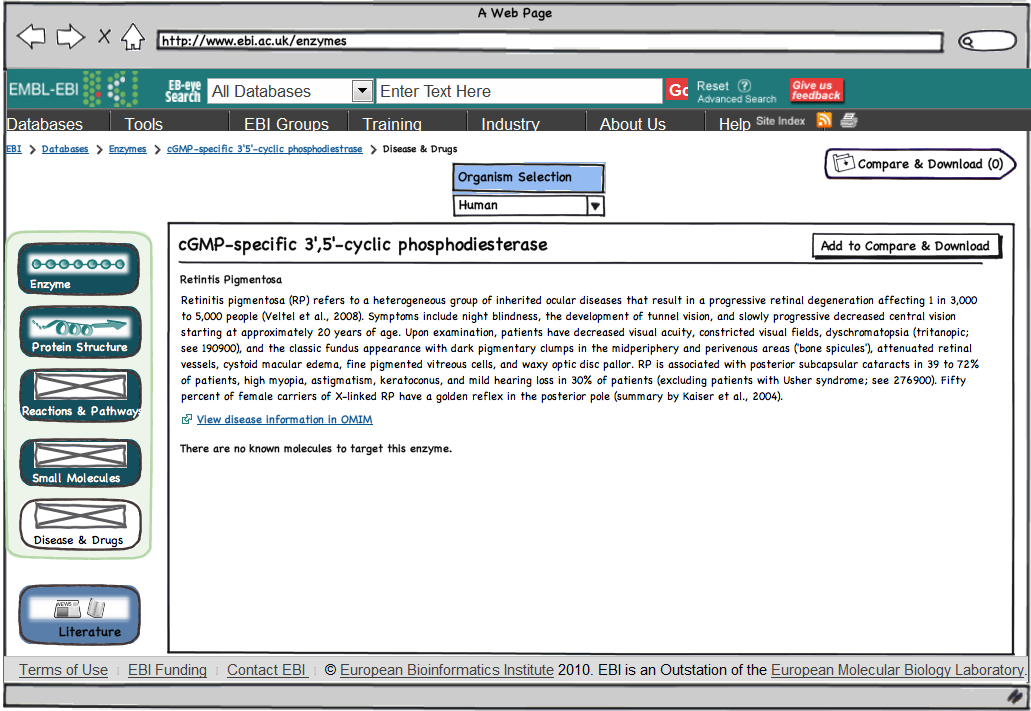

Supplement: Additional file 7 — Balsamiq paper prototypes for usability testing of the Enzyme Portal. [file 1471-2105-14-103-S7.zip › paper prototypes/cGMP Disease and Drugs.png]

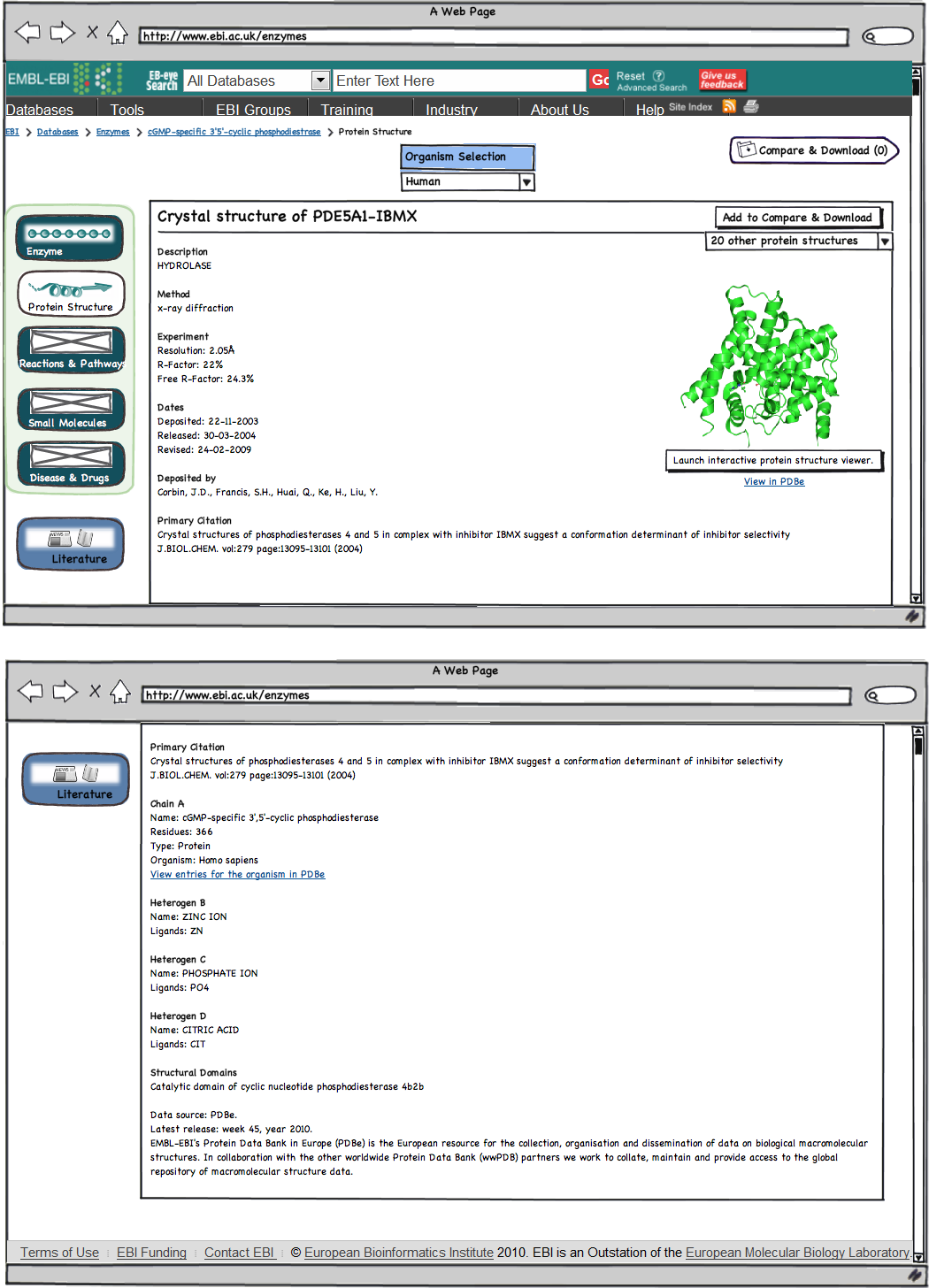

Supplement: Additional file 7 — Balsamiq paper prototypes for usability testing of the Enzyme Portal. [file 1471-2105-14-103-S7.zip › paper prototypes/cGMP Protein Structure.png]

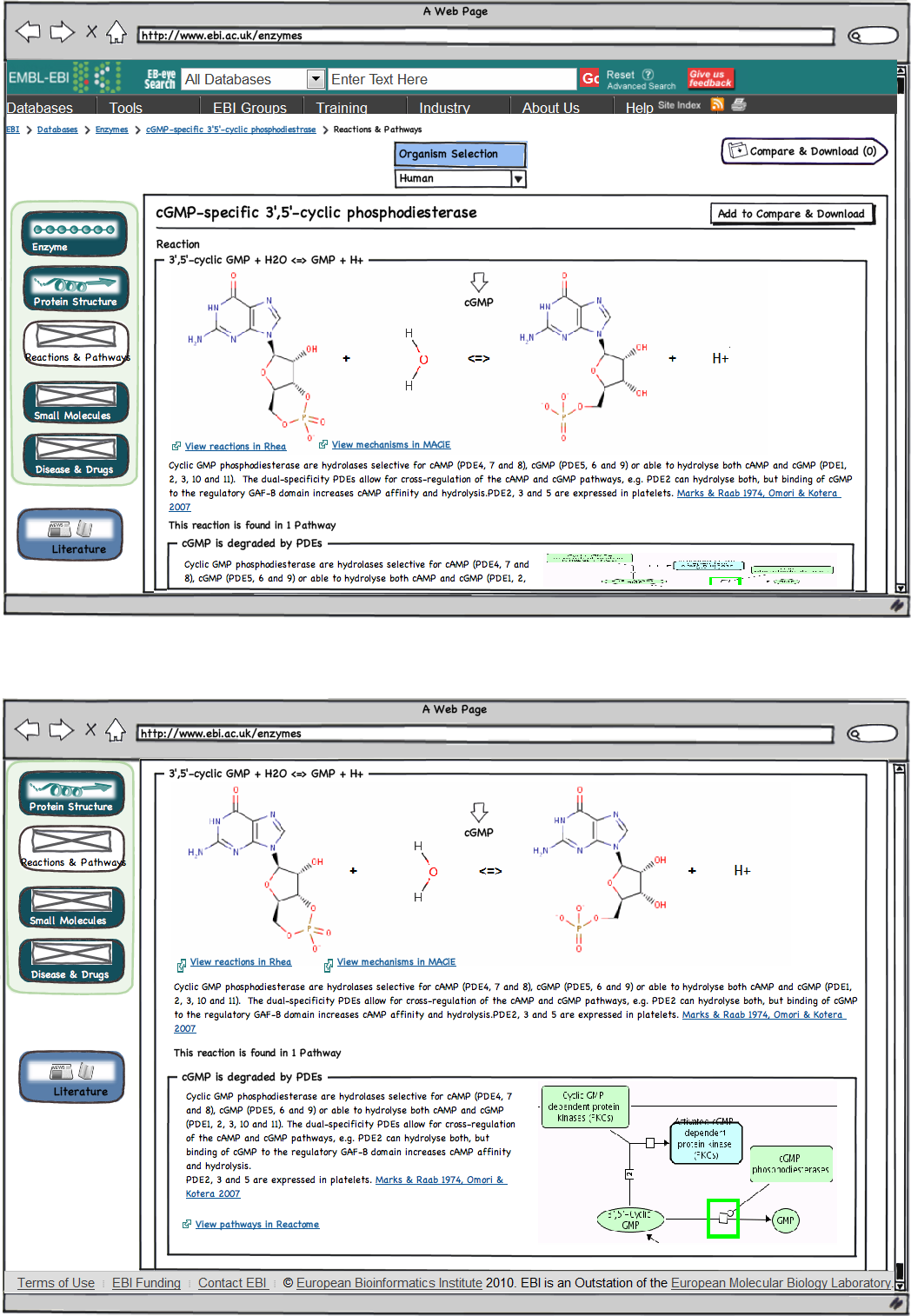

Supplement: Additional file 7 — Balsamiq paper prototypes for usability testing of the Enzyme Portal. [file 1471-2105-14-103-S7.zip › paper prototypes/cGMP Reaction and Pathway Page.png]

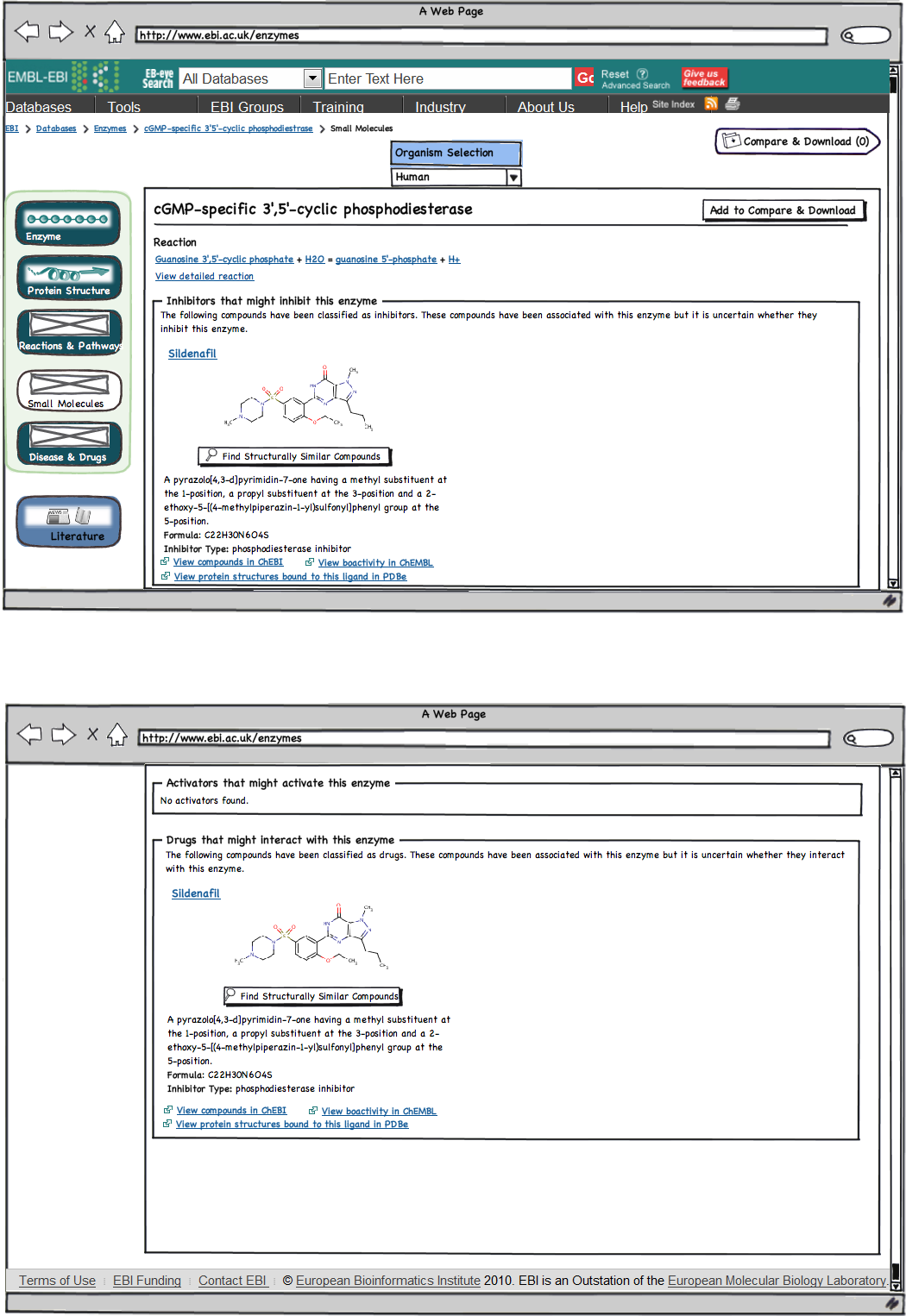

Supplement: Additional file 7 — Balsamiq paper prototypes for usability testing of the Enzyme Portal. [file 1471-2105-14-103-S7.zip › paper prototypes/cGMP Small Molecules Take 2.png]

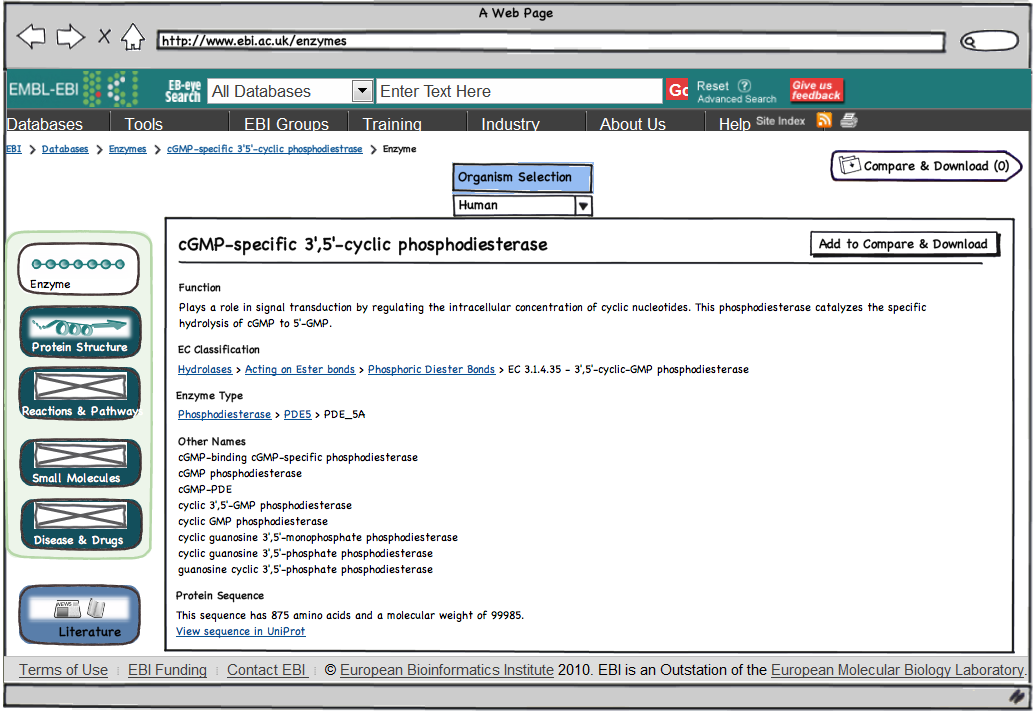

Supplement: Additional file 7 — Balsamiq paper prototypes for usability testing of the Enzyme Portal. [file 1471-2105-14-103-S7.zip › paper prototypes/cGMP Summary Page.png]

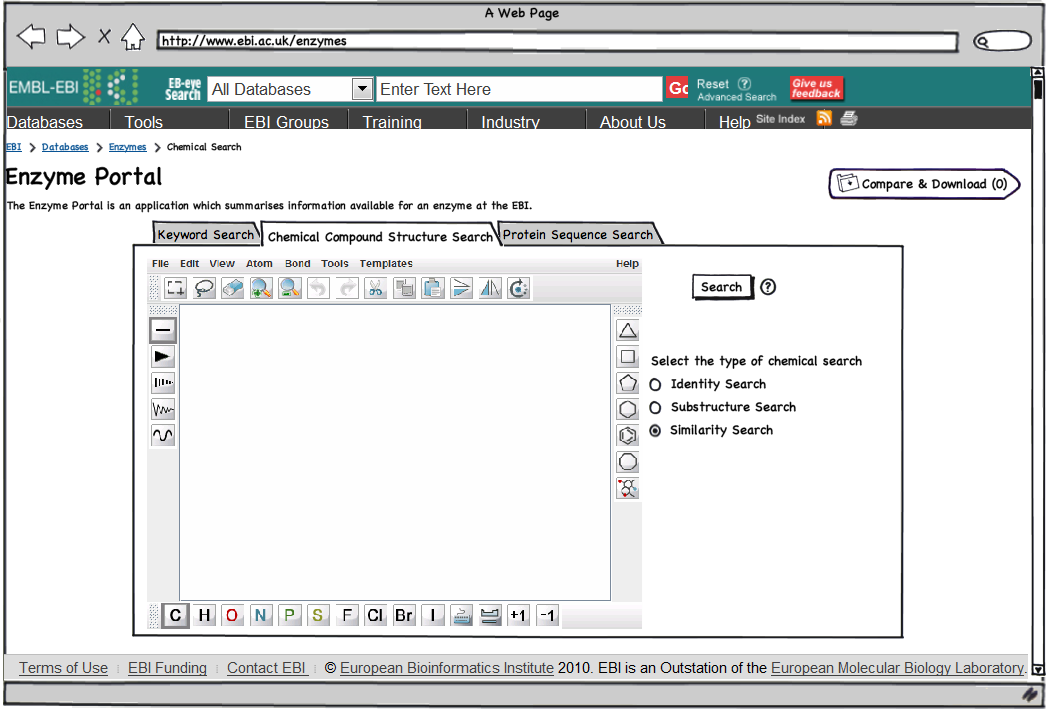

Supplement: Additional file 7 — Balsamiq paper prototypes for usability testing of the Enzyme Portal. [file 1471-2105-14-103-S7.zip › paper prototypes/Chemical Search.png]

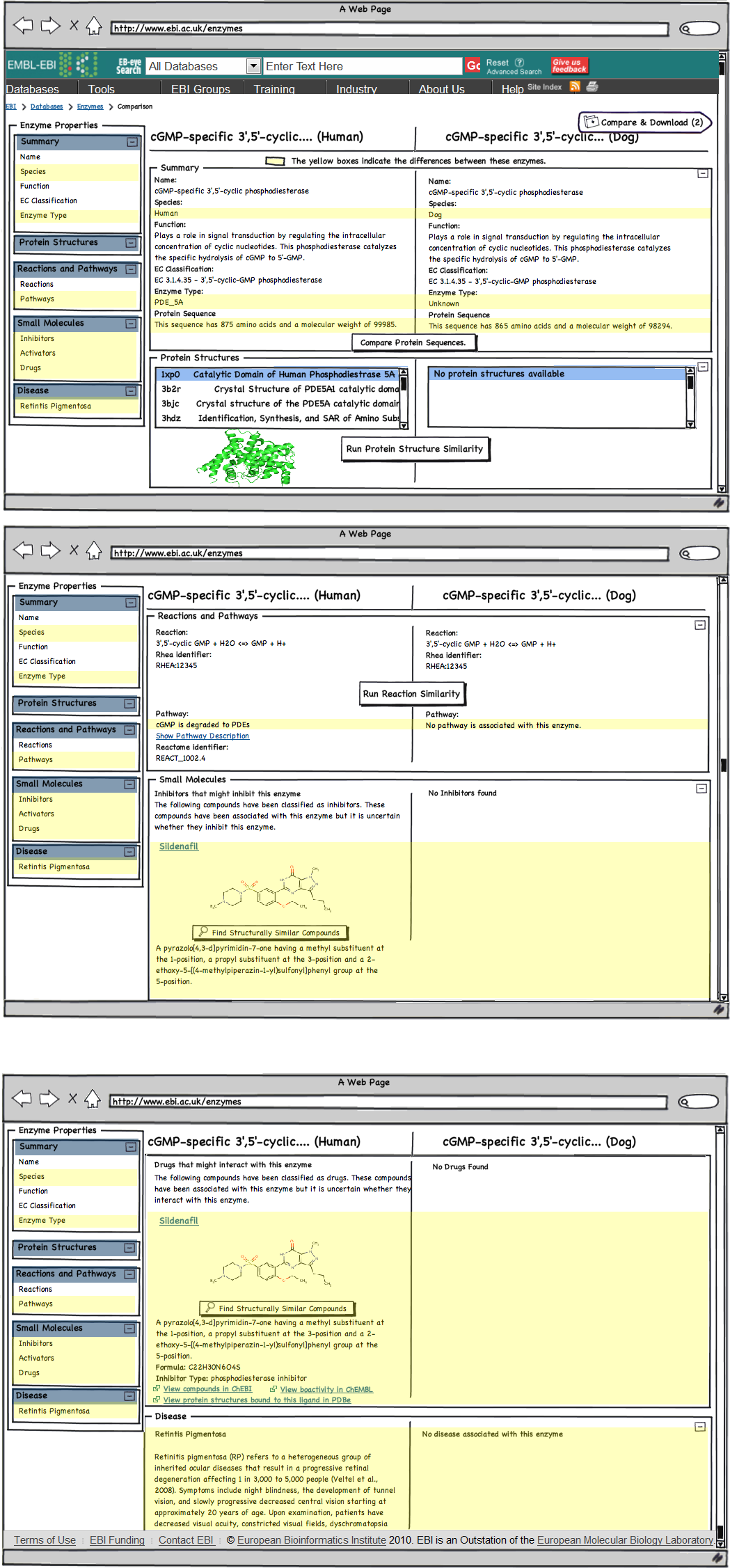

Supplement: Additional file 7 — Balsamiq paper prototypes for usability testing of the Enzyme Portal. [file 1471-2105-14-103-S7.zip › paper prototypes/Enzyme Comparison.png]

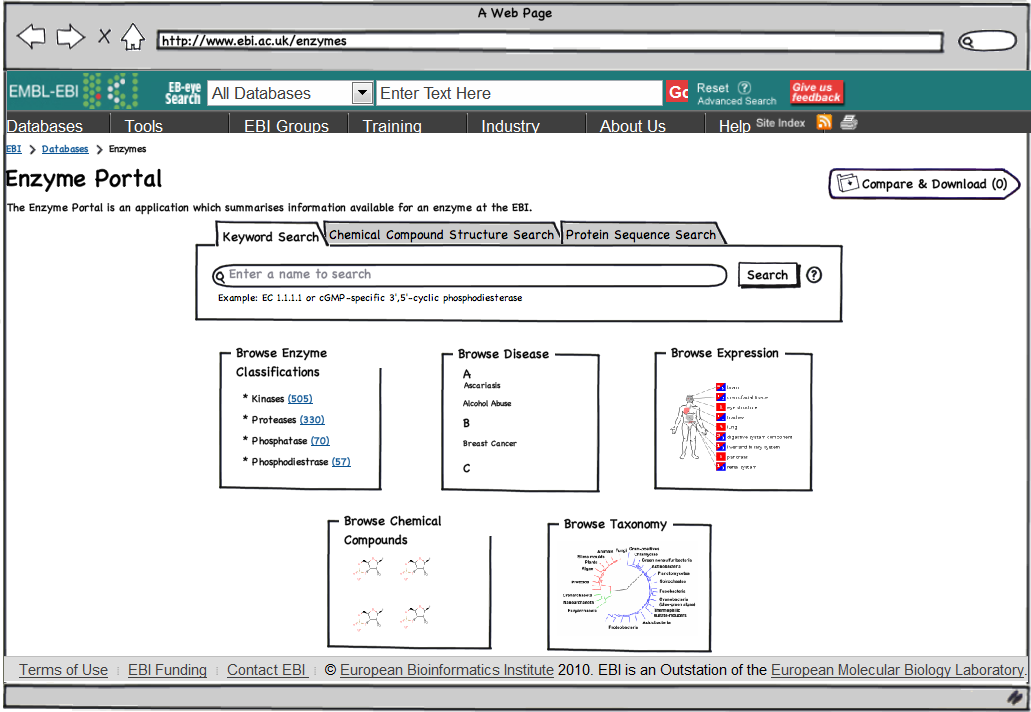

Supplement: Additional file 7 — Balsamiq paper prototypes for usability testing of the Enzyme Portal. [file 1471-2105-14-103-S7.zip › paper prototypes/Home Page.png]

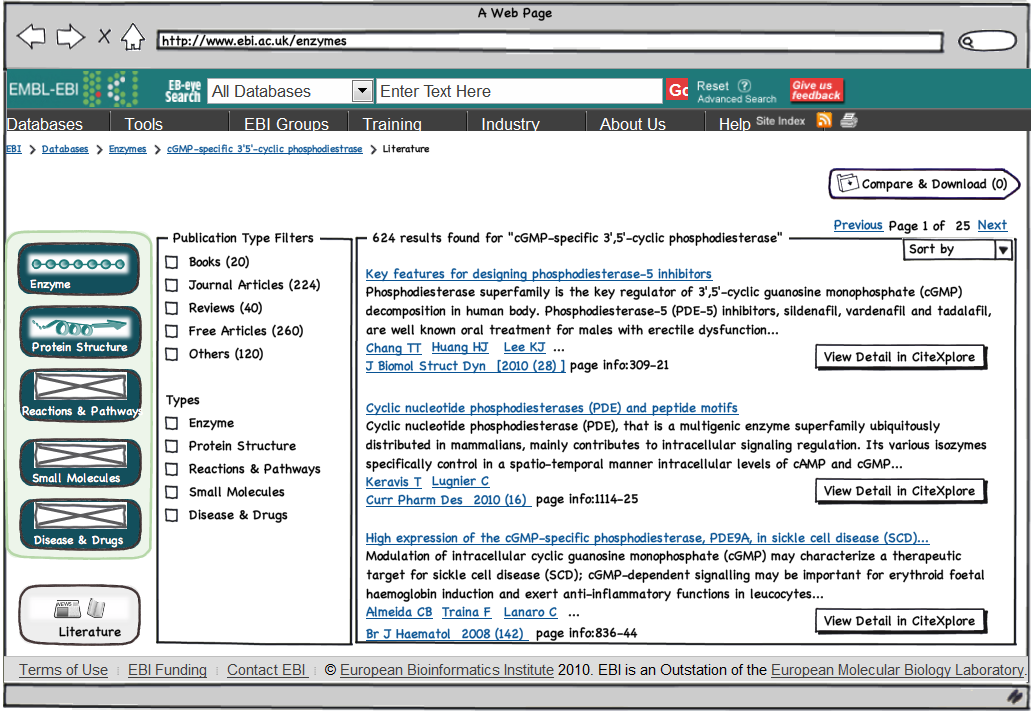

Supplement: Additional file 7 — Balsamiq paper prototypes for usability testing of the Enzyme Portal. [file 1471-2105-14-103-S7.zip › paper prototypes/Literature.png]

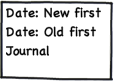

Supplement: Additional file 7 — Balsamiq paper prototypes for usability testing of the Enzyme Portal. [file 1471-2105-14-103-S7.zip › paper prototypes/LiteratureSortBy.png]

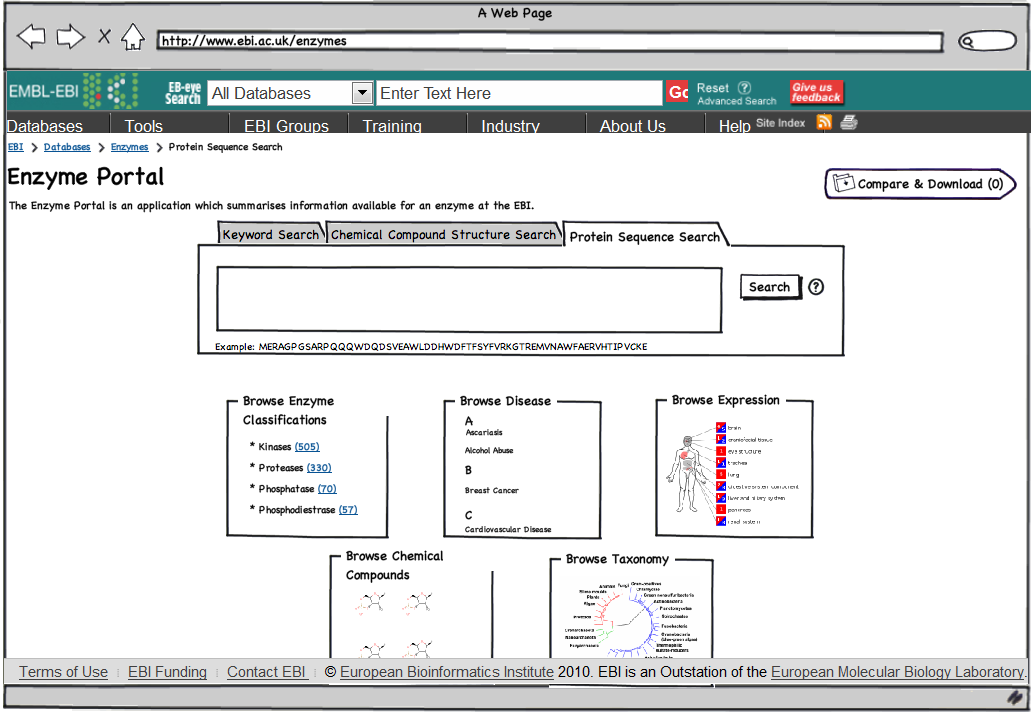

Supplement: Additional file 7 — Balsamiq paper prototypes for usability testing of the Enzyme Portal. [file 1471-2105-14-103-S7.zip › paper prototypes/Protein Sequence Search.png]

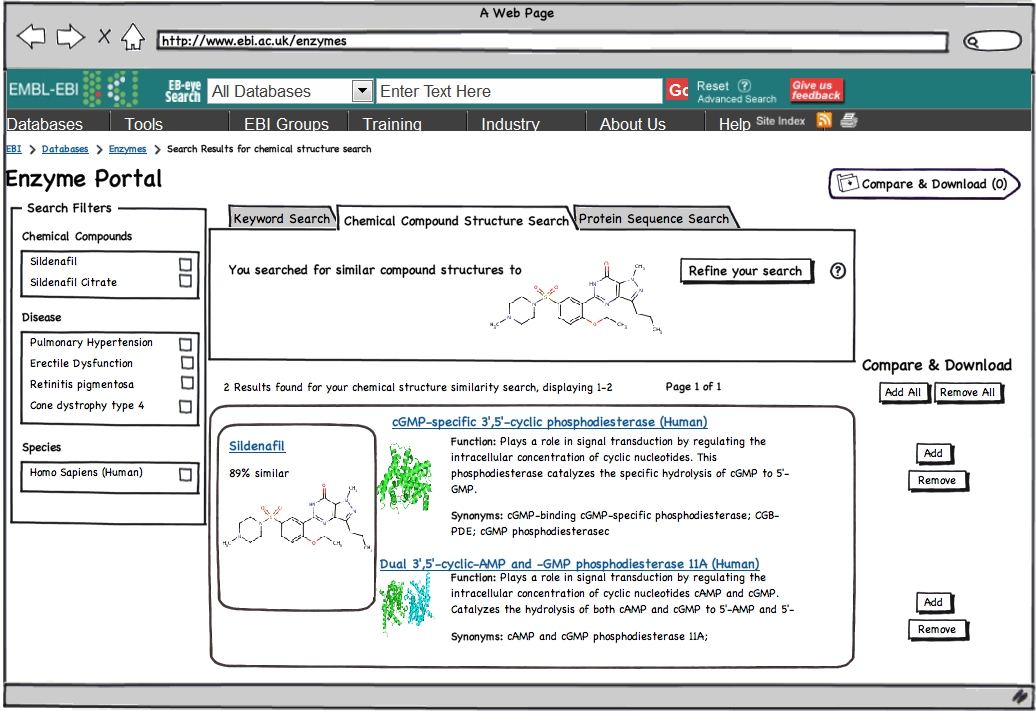

Supplement: Additional file 7 — Balsamiq paper prototypes for usability testing of the Enzyme Portal. [file 1471-2105-14-103-S7.zip › paper prototypes/Search Results Chemical Structure.png]

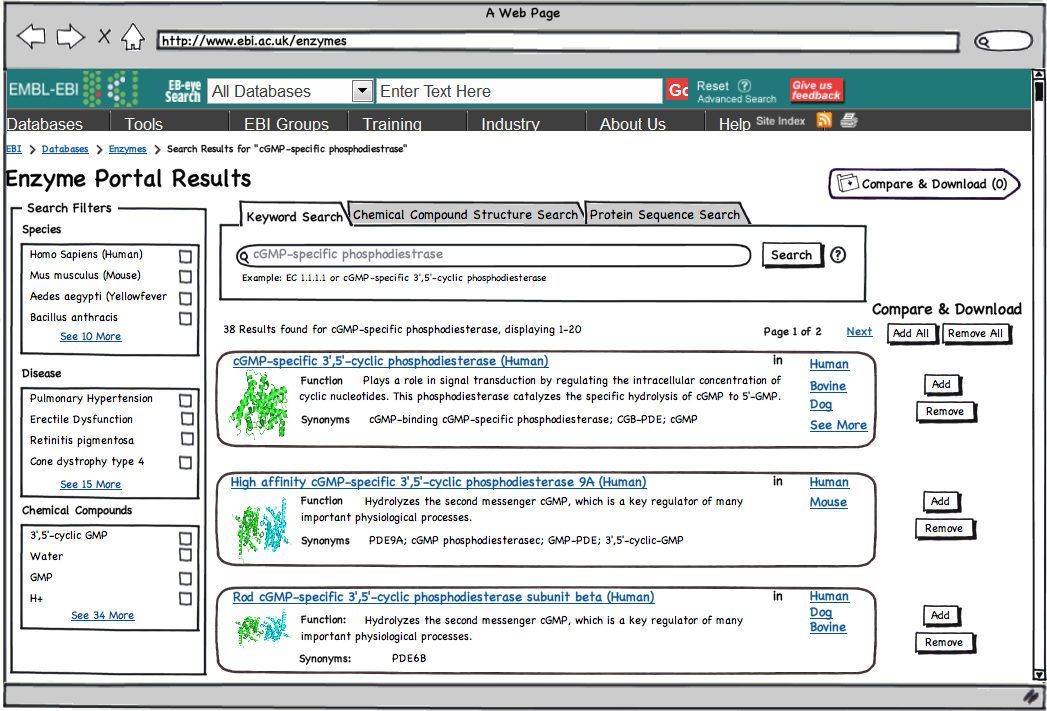

Supplement: Additional file 7 — Balsamiq paper prototypes for usability testing of the Enzyme Portal. [file 1471-2105-14-103-S7.zip › paper prototypes/Search Results Enzyme Name.png]

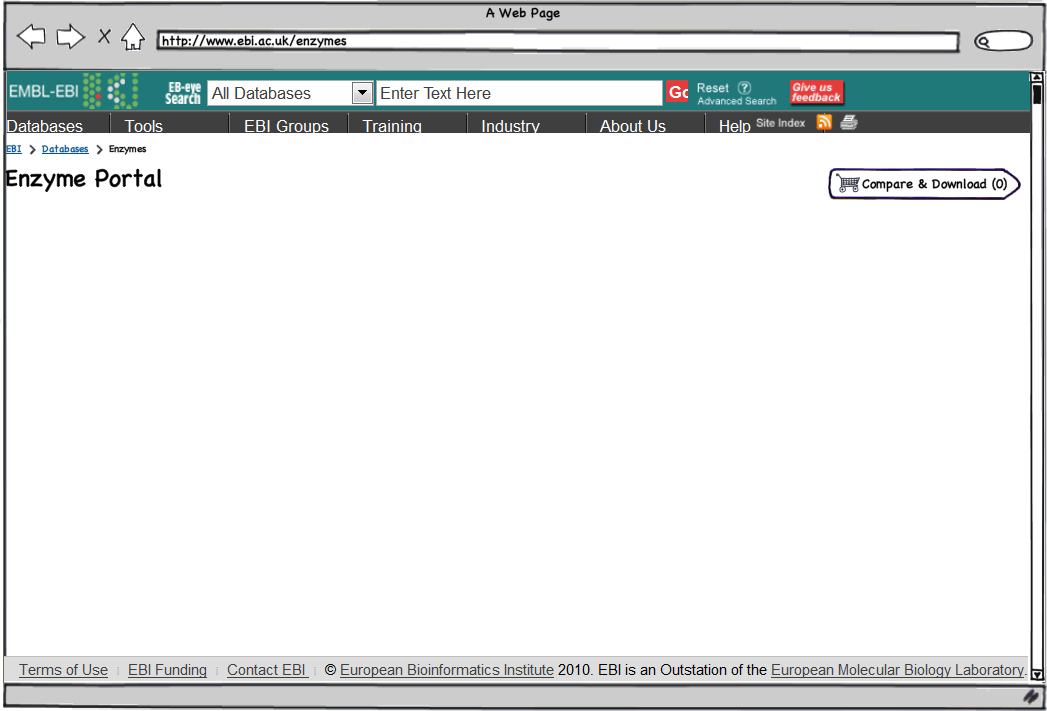

Supplement: Additional file 7 — Balsamiq paper prototypes for usability testing of the Enzyme Portal. [file 1471-2105-14-103-S7.zip › paper prototypes/Under Construction Page.png]
