# Supplementary material for: The Enzyme Portal: a case study in applying user-centred design methods in bioinformatics
Source: BMC Bioinformatics. 2013 Mar 20;14:103. doi: 10.1186/1471-2105-14-103 (PMC3623738; doi:10.1186/1471-2105-14-103)
Supplement: Additional file 2 — Usability study participant consent form. [file 1471-2105-14-103-S2.pdf]

EMBL-EBI

Information sheet for the Enzyme Portal Project

Study: Enzyme Portal Usability Testing

You are being invited to take part in a research study. Please take time to read the following information. Ask us if there is anything that is not clear or if you would like more information. Take as long as you like to decide whether or not you wish to take part.

1. What is the purpose of the study?

This study aims to determine how well the Enzyme Portal interface works for the end user.

2. Why have I been chosen?

You are a key user group we are trying to address.

3. Do I have to take part?

It is up to you to decide whether or not to take part. If you do decide to take part but then decide you wish to withdraw from the study you may do so at any time and without giving reason.
E.g.: If you agree to take part, you will be interviewed or observed for about half an hour. The overall study will take place over several months, but your own input will be relatively brief. The session will be recorded on screen for internal purposes.

The information gathered from your participation will help in understanding the user requirements for this project. No information will be circulated that would make it possible to identify any particular individual's views.

4. Who is organising and funding the research?

This research is being funded by the EMBL-EBI.

5. Contact for Further Information

You can discuss this study with the person who gave you this information sheet, or contact Paula de Matos or Jenny Cham at the European Bioinformatics Institute.

You will be given a copy of the information sheet and a signed consent form to keep.
Thank you for taking part in this study.
Paula de Matos

EMBL-EBI

Study consent form

Identification Number for this trial:

CONSENT FORM

Title of Project: Enzyme Portal Project.

Name of Researcher: Paula de Matos and Jenny Cham

Please initial box

1. I confirm that I have read and understood the information sheet dated.............................. for the above study and have had the opportunity to ask questions. ☐

2. I understand that my participation is voluntary and that I am free to withdraw at any time, without giving any reason, without my legal rights being affected. ☐

3. I agree to take part in the above study. ☐

________________	________________	________________
Participant number	Date	Signature
________________	________________	________________
Researcher	Date	Signature
