# Supplementary material for: The Enzyme Portal: a case study in applying user-centred design methods in bioinformatics
Source: BMC Bioinformatics. 2013 Mar 20;14:103. doi: 10.1186/1471-2105-14-103 (PMC3623738; doi:10.1186/1471-2105-14-103)
Supplement: Additional file 6 — List of data items used in the Canvas Sort. [file 1471-2105-14-103-S6.pdf]

List of data items used in the Canvas Sort

Enzyme nomenclature

EC hierarchy

EC classification

Enzyme Synonyms

Disease

Drug

Toxicology

Residue

Metabolite

Reaction

Molecular interaction

Pathway

Function

Small Molecule

Paper Reference

Species

Organism

Ligand

Cofactor

Inhibitor

Activator

Protein Function

Protein interaction

Sequences

3D structure

Protein domain

Protein expression

Chemical compound

Chemical structure

Species Taxonomy

Fasta Sequence

Substrate

Product

Catalytic role

Active Site

Catalytic mechanism
