# Supplementary material for: The Enzyme Portal: a case study in applying user-centred design methods in bioinformatics
Source: BMC Bioinformatics. 2013 Mar 20;14:103. doi: 10.1186/1471-2105-14-103 (PMC3623738; doi:10.1186/1471-2105-14-103)
Supplement: Additional file 8 — Enzyme portal prototype testing, scenario and tasks. [file 1471-2105-14-103-S8.pdf]

Enzyme Portal Prototype Testing

As you are aware we are developing a service that will integrate the wealth of enzyme data we have at the EBI to enable it to be searchable and understandable.

We have come up with a very basic interface design and would like to test some of its features. We will be giving you a scenario that we would like you to investigate and find out more information about this enzyme. Please think aloud and try to be as verbal as possible about your thoughts. The prototype is designed on paper. Instead of using a mouse you will use a pencil to point and click. The pages are embedded in a paper web browser, you can use the functionality presented on the browser such as scrollbars and back buttons too. Some pages will still not be designed and they will have an "Under Construction Page". I might have to dip into a new page because all the links are not connected. Don't worry we will guide you through it.

We expect this to take about 45 minutes.

Thanks again for your time.

Scenario 1

Sildenafil citrate, sold as **Viagra**, **Revatio** and under various other trade names, is a drug used to treat erectile dysfunction and pulmonary arterial hypertension (PAH). It was developed and is being marketed by the pharmaceutical company Pfizer. It acts by inhibiting cGMP-specific phosphodiesterase type 5, an enzyme that regulates blood flow in the penis. Since becoming available in 1998, sildenafil has been the prime treatment for erectile dysfunction; its primary competitors on the market are tadalafil (Cialis) and vardenafil (Levitra).

Use the Enzyme Portal to discover more information about this enzyme.

Could it be used for example to treat another disease?

Scenario 2

Using two search results above what would you do to see the difference between them?

Questions

These questions are to be asked after the test if they have not already been answered during.

Home Page

Questions:

1. What is your initial reaction when you come to this home page?

2. What would you expect to see here?

3. Can you clearly find the search box?

4. Browse functionality obvious?

5. Can you see the different search options?

Search Results Page

Note: Write down possible search terms.

6. What would you expect to put in the search box?

7. When you search for a specific term – what result do you expect e.g. caffeine?

8. Have you noticed the left hand box. Is it obvious what to do there?

Enzyme Entry Page

9. What name would you use to associate all the data presented to you e.g. Metabolism?

10. If a tRNA is in the reaction of an enzyme, can you describe where the information would go?

11. What does Favourites mean to you?

Comparison

1. What features did you find useful?

2. Was it obvious to get here?

After the test

Which functionality available on the Home Page would you most obviously use?

Did the site do what you expected?
